# Genome-wide analysis of human hotspot intersected genes highlights the roles of meiotic recombination in evolution and disease

**DOI:** 10.1186/1471-2164-14-67

**Published:** 2013-01-31

**Authors:** Tao Zhou, Zhibin Hu, Zuomin Zhou, Xuejiang Guo, Jiahao Sha

**Affiliations:** 1 State Key Laboratory of Reproductive Medicine, Nanjing Medical University, 140 Hanzhong Road, Nanjing, Jiangsu Province, 210029, People’s Republic of China

**Keywords:** Meiotic recombination, Recombination hotspots, Evolution, Disease, Chromosomal rearrangements, Repeat element

## Abstract

**Background:**

Meiotic recombination events are not randomly located, but rather cluster at hotspot regions. Recently, the fine-scale mapping of genome-wide human recombination hotspots was performed. Here, we systematically analyzed the evolutionary and disease-associated features of hotspots that overlapped with protein-coding genes.

**Results:**

In this study, we defined hotspot intersected genes as HI genes. We found that HI genes were prone to be located in the extracellular part and were functionally enriched in cell-to-cell communication. Tissue-specific genes and secreted protein encoding genes were overrepresented in HI genes, while housekeeping genes were underrepresented. Compared to slowly evolving housekeeping genes and random genes with lower recombination rates, HI genes evolved faster. The fact that brain and blood specific genes were overrepresented in HI genes indicates that they may be involved in the evolution of human intelligence and the immune system. We also found that genes related to disease were enriched in HI genes, especially genes with disease-associated chromosomal rearrangements. Hotspot sequence motifs were overrepresented in common sequences of HI genes and genes with disease-associated chromosomal rearrangements. We further listed repeat elements that were enriched both in hotspots and genes with disease-associated chromosomal rearrangements.

**Conclusion:**

HI genes are evolving and may be involved in the generation of key features of human during evolution. Disease-associated genes may be by-products of meiotic recombination. In addition, hotspot sequence motifs and repeat elements showed the connection between meiotic recombination and genes with disease-associated chromosomal rearrangements at the sequence level. Our study will enable us to better understand the evolutionary and biological significance of human meiotic recombination.

## Background

Meiotic homologous recombination is necessary for accurate chromosomal disjunction which is important in maintaining genome integrity. It also contributes to genetic diversity by generating new combinations of alleles, thereby providing raw materials for evolution [1,2]. Errors in meiotic recombination can cause genome instability and diseases. For example, chromosome nondisjunction or reduced recombination may result in constitutive aneuploidy, which can lead to spontaneous abortion or congenital birth defects. In addition, non-allelic homologous recombination (NAHR) can cause chromosomal rearrangements, many of which have been associated with diseases [3]. To ensure accurate homologous recombination during meiosis, the location and rate of recombination should be precisely regulated. Previous studies have found that meiotic recombination events are non-randomly located, but cluster at hotspots that exhibit elevated rates of recombination [4]. Recent studies have constructed high-resolution fine-scale maps of recombination rates and hotspots across the human genome [5]. In addition, hotspot-associated sequence motifs have also been reported in the human genome [6]. The PR domain zinc finger protein 9 (PRDM9) is thought to be a transregulator of meiotic recombination hotspots in humans and mice, and the hotspot sequence motifs may serve as binding sites for this protein [7]. However, the motifs of hotspot may also drive genome instability, as they are found in disease-causing repeat sequences and breakpoint regions [8]. Since hotspots are not shared between humans and chimpanzees, hotspot sequences are thought to have evolved quickly [9].

In the present study, we focused on the consequences of human meiotic recombination hotspots, in regard to determining their effect on genome evolution, and evaluating their association with genome instability and diseases. In order to answer these questions at the genome and protein-coding gene levels, we first defined the human meiotic recombination hotspot intersected gene as the HI gene. Since HI genes directly overlapped with recombination hotspots, they may reflect the features and consequences of recombination. We first analyzed the functional enrichments of HI genes, and then studied the evolutionary features of HI genes by comparing them to slowly and quickly evolving genes. We also analyzed the association of HI genes with human diseases and sought to explore the sequence basis for the connections. These findings will enable us to better understand the evolutionary and biological significance of human meiotic recombination hotspots.

## Results and discussion

### General features of HI genes

There were 2,054 transcripts that intersected with 1,775 hotspots. In total, we obtained a list of 1,156 hotspot intersected genes, and named the dataset HI genes. The transcripts of HI genes were merged to 1,136 non-overlapping regions. The minimum, maximum, and median lengths of HI gene regions that were covered by hotspots are 7 bases, 120,000 bases, and 10,000 bases respectively. About 72% of HI gene regions covered at least one entire hotspot region, and about 97% of HI gene regions covered at least 1,000 bases with hotspot regions.

### Functional annotation of HI genes

**Figure 1:**
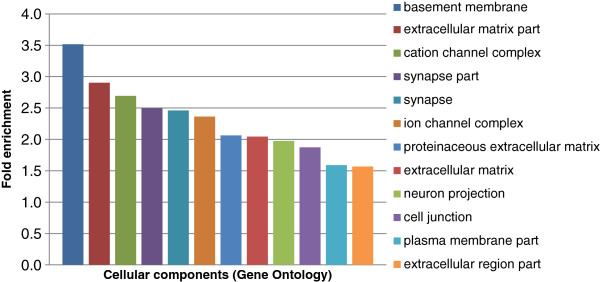
**Enriched cellular components of HI gene products.** The horizontal axis represents enriched classes of cellular components and the vertical axis represents the fold enrichment. Cellular components are colored and annotated to the right.

Gene Ontology analysis showed that HI gene products were prone to be located in the extracellular matrix, ion channel complex, plasma membrane part, and cell junctions (Figure 1; Table S1 in Additional file 1), indicating that HI genes may play an important role in cell-to-cell cross-talk and the paracellular barrier. HI genes were also enriched in the nervous system at locations including the neuron projection and synapse. Enriched molecular functions revealed that HI gene products had significantly channel activity. Enriched biological processes also revealed that HI genes were mainly involved in cell adhesion, cell morphogenesis, ion transport, and neuron development.

Previous study have shown that hotspot predictions are enriched at genes with particularly important roles in the central nervous system [10]. Our results, based on fine-scale mapping of the recombination rate, also provided evidence that HI genes were preferentially located around ion channels and are involved in neuron development. Cell adhesion and extracellular components are important for the evolution of multicellular organisms [11]. Since HI gene products were enriched in cell adhesion and the extracellular matrix, we hypothesized that HI genes may play roles in human evolution.

### HI genes and human evolution

**Figure 2:**
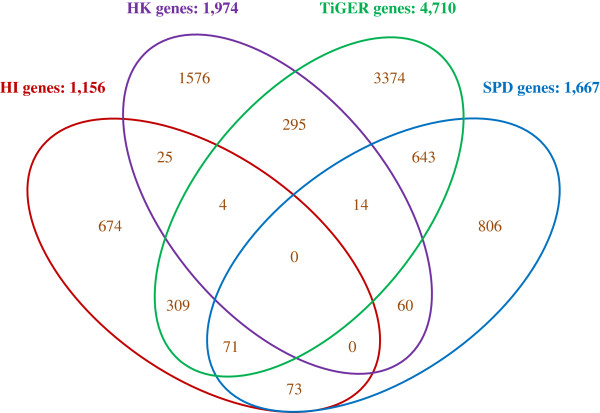
**Venn diagram of the overlap between and among four datasets.** Gene datasets are colored according to gene classes (HI genes, HK genes, TiGER genes and SPD genes), and the total gene number is shown beside the dataset name. The unique and shared gene numbers between and among the four gene datasets are shown in the unique and overlapped regions.

Tissue-specific genes are relatively highly expressed in one or several tissues or cell types, while housekeeping genes are generally ubiquitously expressed in all cells. In regard to evolution, housekeeping genes have evolved more slowly than tissue-specific genes [12]. Secreted proteins are secreted from cells into the extracellular space, and play important regulatory roles in multicellular organisms. They also evolve at faster rates than non-secreted proteins, and their evolutionary rates are highly correlated with tissue specificity [13]. To analyze the evolutionary features of HI genes, we first compared the overlap of housekeeping genes, tissue-specific genes, genes encoding secreted proteins and HI genes (Figure 2). We found that housekeeping (HK) genes were significantly underrepresented in HI genes (FE = −4.3; P < 0.001); only 29 HK genes were found in HI genes. However, genes from TiGER, a dataset for tissue-specific gene expression and regulation, were enriched in HI genes (FE = 1.3, P < 0.001), and genes from the secreted protein database (SPD) were also enriched in HI genes (FE = 1.4, P < 0.01). This indicates that HI genes may share more feature with SPD genes and TiGER genes, but are different from HK genes in evolution.The fact that SPD genes and TiGER genes were overrepresented in HI genes, indicates that HI genes may have also evolved more quickly than housekeeping genes. To test this hypothesis, we compared the dN (the number of non-synonymous substitutions per non-synonymous site), dS (the number of synonymous substitutions per synonymous site), and dN/dS values of HK genes, TiGER genes, and SPD genes with HI genes (Table 1; Tables S2 and S3 in Additional file 1). The evolutionary rates of HI genes were significantly greater than HK genes, and less than TiGER genes and SPD genes. It appears that HI genes located in the hotspots of recombination evolved faster than slowly evolving housekeeping genes. Since the dN/dS values of HI genes were greater than the housekeeping genes, HI genes might be under less selective constraints than housekeeping genes. We next compared the evolutionary rates of HI genes to MI genes (located in the regions with middle recombination rates) and CI genes (located in the regions with low recombination rates). Both MI genes and CI genes had lower evolutionary rates than HI genes (Table 1; Tables S2 and S3 in Additional file 1). The dN, dS, and dN/dS values of CI genes were significantly less than HI genes, indicated that genes located in hotspot regions evolved faster than other regions with lower recombination rates.

**Table 1:** Comparison of evolutionary rates

**Dataset**	**dN**	**dS**	**dN/dS**
HK	- (***)	- (***)	- (***)
TiGER	+ (***)	+ (***)	+ (***)
SPD	+ (***)	+ (***)	+ (***)
MI	- (**)	- (***)	-
CI	- (***)	- (***)	- (**)

The dN, dS and dN/dS values of HK genes, TiGER genes, SPD genes, MI genes and CI genes are compared to HI genes. The symbol ‘-’ indicates the median value is less than HI genes and ‘+’ indicates the median value is greater than HI genes. The symbol ‘***’ indicates a p value less than 0.001 and ‘**’ indicates a p value less than 0.01.

The results of the evolutionary rates were consistent with previous findings demonstrating that human SNP (single-nucleotide polymorphism) variability and mutation rate are higher in regions of high recombination [14]. The faulty repair of DSBs (double-strand breaks) that initiated recombination could explain the mutagenesis of recombination. However the dN/dS values of HI genes, MI genes, and CI genes were really close numerically (Table S2 in Additional file 1). This could be explained by the viewpoint of historical recombination rates. Humans and common chimpanzees share about 95 ~ 99% of their DNA (deoxyribonucleic acid), but hotspot locations are markedly different, and the sex averaged recombination rates of humans might be elevated relative to other primates [1]. It seems that hotspots themselves were evolving, and some regions of hotspots might shift during evolution. The current hotspots in human were observed, but there may be historical hotspots in other regions. So the already evolved historical HI genes may also displayed relative elevated evolutionary rates.

Tandem duplicated genes are mainly generated by ‘unequal crossing over’, which results from homologous recombination between paralogous sequences [15]. To further analyze the evolutionary consequences of recombination hotspots, we calculated the proportions of DGD (Duplicated genes) [16] transcripts in HI transcripts, MI transcripts, CI transcripts, HK transcripts, TiGER transcripts, SPD transcripts, and hg18rpa (RefSeq protein-coding genes located in autosomal chromosomes, based on human genome hg18) transcripts (Table S4 in Additional file 1). We found that HI transcripts had a higher percentage of DGD transcripts than HK transcripts (20.1% versus 9.1%, P < 0.0001), indicating that HK genes are very different from HI genes in evolution. However compared to HI transcripts, hg18rpa transcripts had a similar percentage of DGD transcripts (22.2%, P > 0.05), and both TiGER transcripts and SPD transcripts had a significantly higher percentage of DGD transcripts than HK transcripts.

As the whole protein-coding genes had a high percentage of duplicated genes, this hinted at the shift of hotspot regions during evolution. MI transcripts and CI transcripts had a lower percentage of DGD transcripts than HI transcripts; however TiGER transcripts and SPD transcripts had a higher percentage of DGD transcripts. These results were consistent with the evolutionary rates of HI genes, HK genes, TiGER genes, SPD genes, MI genes, and CI genes. Thus we concluded that genes located in recombination regions are currently evolving.

**Figure 3:**
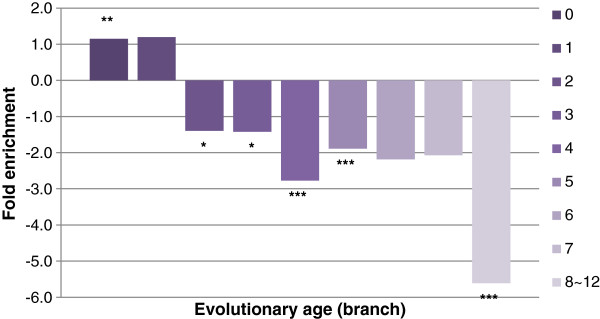
**Fold enrichments of HI genes in different evolutionary ages.** The horizontal axis represents evolutionary age and the vertical axis represents the fold enrichment. Values are sorted by evolutionary branch. Branch 0 indicates the oldest genes and branches 8 ~ 12 indicate primate-specific young genes. Significantly enriched branches are labelled with asterisks (*: P < 0.05; **: P < 0.01; ***: P < 0.001).

Previous studies have classified human genes into different evolution ages [17]. We analyzed the distribution of HI genes in AGE (gene dataset annotated with different evolutionary age) genes. About 79.8% HI genes were the oldest genes (evolutionary branch 0). We found that the very old genes (branch 0 and 1) were overrepresented in HI genes, but other branches of genes including primate-specific young genes (branches 8 ~ 12) were underrepresented in HI genes (Figure 3). New human genes mainly evolve from old genes, and highly correlate with recombination and duplication [18]. Our results indicated that most HI genes were old genes that were still evolving, and that still provide materials for the evolution of new genes by their high frequency of recombination. HI genes may represent the present and the future of human revolution. We further analyzed the distribution of HI genes in different tissues using TiGER genes. The TiGER dataset contains tissue-specific genes for 30 human tissues. We found that TiGER genes in 10 tissues included brain, blood, soft tissue, colon, kidney, eye, lymph node, spleen, tongue, and mammary gland were significantly enriched in HI genes (Table S9 in Additional file 1). These tissues may also be evolving as HI genes were evolving. Tissues such as brain and blood represented the intelligence and immune system of human which are different than other primates [19,20]. As tissue-specific genes of brain were enriched in HI genes, we inferred that HI genes may be involved in the generation of key features of humans during evolution.

### HI genes and human diseases

**Figure 4:**
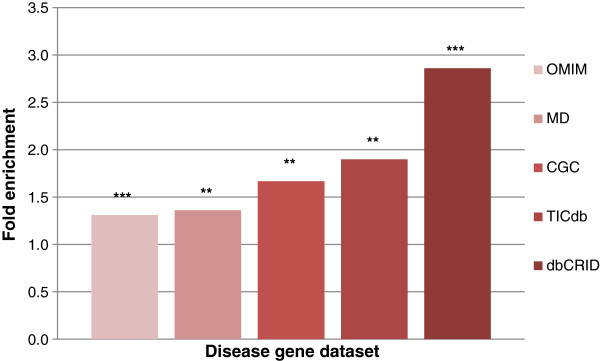
**Fold enrichments of disease-associated genes in HI genes.** The horizontal axis represents the disease gene dataset and the vertical axis represents the fold enrichment. Values are sorted by fold enrichment. Statistical significance is labelled with asterisks (**: P < 0.01; ***: P < 0.001).

To systemically study the association of HI genes and diseases, we determined the enrichment of disease-associated genes in HI genes. We found that all diseases-associated gene datasets including OMIM (Mendelian disorder-associated gene dataset) genes, MD (Mendelian disease gene dataset with at least one mutation in the particular gene is causative of the disease) genes, CGC (cancer gene dataset) genes, TICdb (reciprocal translocation associated gene dataset in human tumours) genes, and dbCRID (chromosomal rearrangement associated gene dataset in human diseases) genes were significantly enriched in HI genes (Figure 4). Among these datasets, OMIM genes had the lowest value of fold enrichment (FE = 1.3), and dbCRID genes had the highest value of fold enrichment (FE = 2.9). Additionally, we constructed a dataset called InteCR (integrated of chromosomal rearrangement associated disease genes in dbCRID, TICdb, and CGC genes). We found that InteCR genes were also enriched in HI genes (FE = 2.4, P < 0.001; Table S9 in Additional file 1).

The OMIM dataset includes genes associated with a mixture of all kinds of human disorders, and MD genes were also extracted from OMIM genes but were filtered to include only those genes with strong evidence showing that at least one mutation in the particular gene is causative of disease. MD genes are reported to have a tendency to be ancient genes that are highly associated with duplication events during evolution [21]. The enrichment of MD genes in HI genes further indicated that heritable diseases may be by-products of the evolutionary process.

The dbCRID dataset contains genes involved in chromosomal rearrangement in human diseases [22]. Most part of dbCRID genes are those genes involved in reciprocal translocation (71.0% in dbCRID genes, 76.7% in HI-dbCRID common genes), and reciprocal translocation associated dbCRID genes were enriched in HI genes (FE = 3.09, P < 0.001). Interestingly, TICdb genes also had a higher value of fold enrichment than CGC genes. CGC genes are consisting of all mutated genes in human cancer [23]. In terms of mutation type, most CGC genes are translocation associated genes (66.5% in CGC genes, 88.9% in HI-CGC common genes); translocation associated CGC genes were also enriched in HI genes (FE = 2.23, P < 0.001). The TICdb genes, which contain reciprocal translocation associated genes in human tumours, were also enriched in HI genes. Together, these results show that chromosomal rearrangements in human diseases are highly associated with recombination hotspots. For example, BCL2 is annotated as an HI gene and is located on 18q21.33. BCL2 was initially reported to be involved in t(14;18) translocation in lymphoma [24]. Reed JC et al. discovered the oncogenic potential of BCL2 by gene transfer [25]. Another example of an HI gene is RAD51, which is located on 14q24.1. RAD51 plays key roles in both mitotic and meiotic homologous recombination and in DNA double-strand break repair. A region of RAD51 is reported to be involved in reciprocal translocations in uterine leiomyomas [26].

There were only 29 genes found both in HI-MD (20.6%) and HI-InteCR common genes (31.5%), indicating that genes involved in heritable diseases and chromosomal rearrangements associated diseases are to some extent two different classes of genes. MD genes are disease-causing genes, but may not directly associate with recombination events. Thus, some Mendelian heritable diseases may be indirect consequences of recombination events during evolution. However, InteCR genes are involved in disease causing chromosomal rearrangements at the sequence level, and corresponding chromosomal rearrangements may be directly associated with recombination events. Both homologous recombination and non-homologous end joining are the main repair systems for handling DNA double-strand breaks [27]. Actual crossover events that would result in genome rearrangements are found to be suppressed during the recombinational repair of double-strand breaks [28]. Thus meiotic recombination does not aim to cause diseases, but we did observe that many diseases genes were enriched in HI genes. As HI genes were directly overlapped with meiotic recombination hotspot, we explained it by the fact that some diseases may be by-products of faulty recombination events in meiotic recombination hotspot regions.

Meiotic non-allelic homologous recombinations in the germ line can cause genome instability, and thus result in genomic disorders, but the associated protein-coding genes are not well annotated [3]. The most frequent chromosomal rearrangement associated disease is cancer [22], and the most frequent type in cancer is translocation [23]. About 90.8% CGC genes show somatic mutations (the corresponding percentage for HI-CGC common genes is 97.8%). HI genes were extracted from meiotic recombination hotspots, but chromosomal rearrangement associated genes in somatic disease were enriched in HI genes. Mitotic recombination has been reported to be associated with carcinogenesis [29]. However, hotspots for mitotic recombination in human have not been fully studied. Our results suggest that there may be some connections between meiotic and mitotic recombination hotspots at the sequence level.

**Figure 5:**
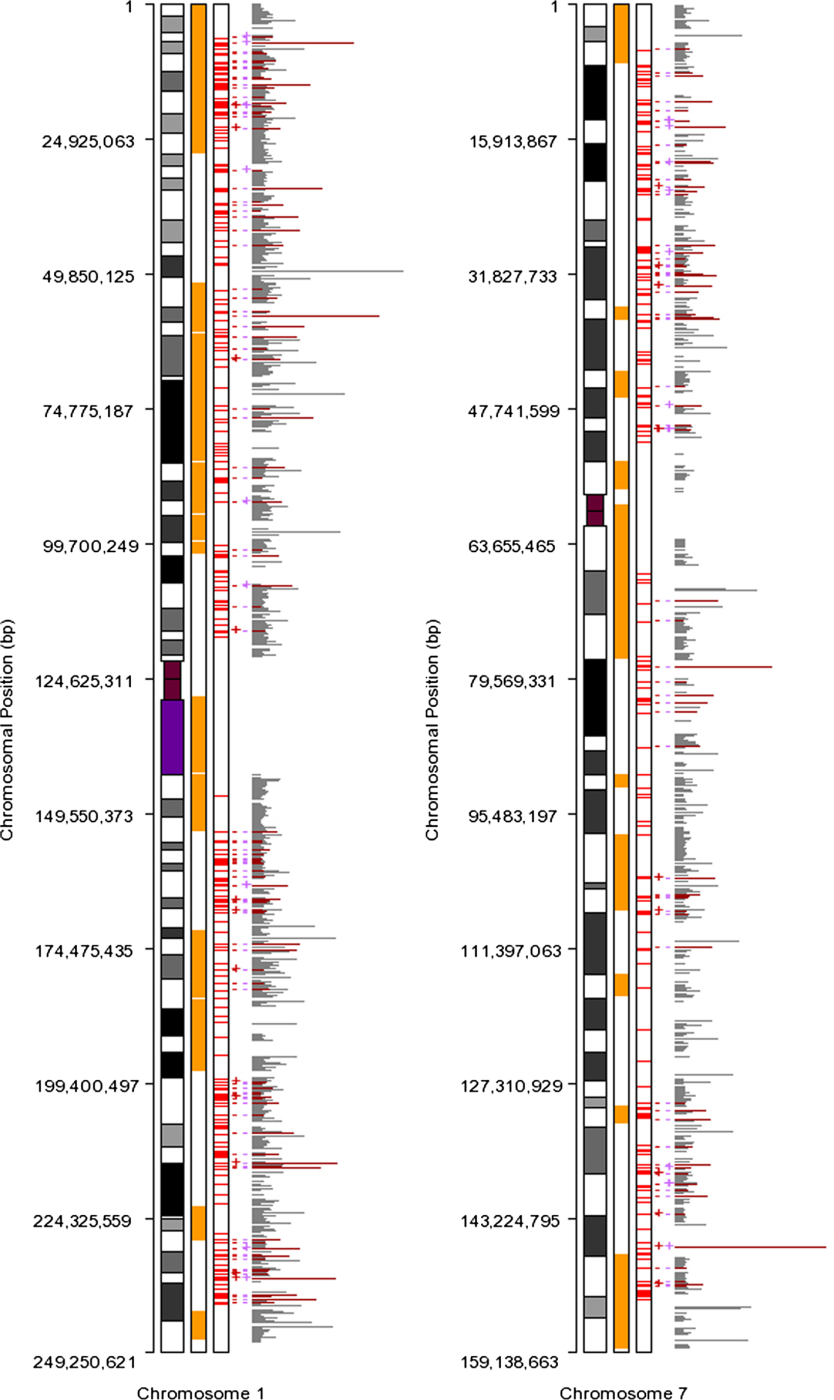
**Representative integrated chromosomal maps for chromosome 1 and 7.** For each map, the horizontal axis in the leftmost represents chromosome length. The first rectangle stands for chromosomal ideograms. Each orange region in the third rectangle stands for a fragile site location. Each red line in the second rectangle stands for a hotspot. The gray lines in the rightmost part stand for hg18rpa genes. The length of each line reflects the relative length of a gene. HI genes are colored red and annotated to the left with '-' (no) or ' + ' (yes) based on gene types. Red and purple stand for MD gene and InteCR gene respectively.

Fragile sites are prone to chromosomal double-strand break (DSB), and associated with mitotic recombination [30]. Currently there are 114 fragile sites on autosomal chromosomes [31]. A total of 89 (78.1%) fragile sites are overlapped with 662 (32.2%) HI transcripts. This indicates the chromosomal fragility of HI genes. Representative chromosomal maps for fragile sites, hotspots, HI genes, MD genes, and InteCR genes are shown in Figure 5. Full maps are given in Additional file 2.

To further explore the common molecular features of recombination hotspots and genes involved in chromosomal rearrangements associated diseases, we analyzed the occurrences of hotspot motifs and repeat elements in HI gene regions and InteCR gene regions. There are three sequence motifs including CCTCCCT, CCCCACCCC and CCNCCNTNNCCNC associated with recombination hotspots [8]. All these motifs had elevated proportions in InteCR gene regions, HI gene regions, and HI-InteCR common gene regions compared to hg18rpa gene regions (Table S5 in Additional file 1). The most significantly enriched motif was CCCCACCCC. That indicates the motif CCCCACCCC is mostly responsible for chromosomal rearrangements associated diseases. We found 130 repeat elements which were significantly overrepresented in hotspot regions compared to autosomal genome sequences (Table S6 in Additional file 1). Previously reported enriched repeat elements in hotspot sequences including THE1B, THE1A, CT-rich, and L2 are also in the list [8]. These repeats may drive recombination regions to hotspots. To further search possible repeats that are responsible for chromosomal rearrangement associated diseases, we analyzed the occurrences of repeat elements in HI-InteCR gene regions. There were 51 repeat elements enriched in HI-InteCR gene regions compared to InteCR gene regions, and 37 of them were also enriched repeat elements in hotspot regions (Tables S7 and S8 in Additional file 1). These 37 common repeat elements may play a key role in both driving recombination hotspots and chromosomal rearrangement associated diseases. For example, MIRb is one of the enriched repeat elements. MIRb insertion was reported to be involved in paraganglioma and pheochromocytoma which are associated with germline mutation of the tumour suppressor genes [32].

## Conclusions

Meiotic recombination is important for evolution and is also highly associated with genome instability. Since HI genes are overlapped with recombination hotspots, HI gene regions may also endure recombination at high frequency. We systematically analyzed the evolutionary and biomedical features of meiotic recombination hotspots at the protein-coding gene level. Thus, it enables us to better understand the consequences of recombination during evolution since the protein-coding genes are well annotated functionally. Our results illustrate the double sides of meiotic recombination.

### The evolutionary importance of meiotic recombination

Our results demonstrate that HI genes are functionally enriched in cell-to-cell communication which is a key function for multicellular organisms. Compared to slowly evolved housekeeping genes, HI genes evolved more quickly. We also believe that hotspots themselves are changing during evolution. As most HI genes are old genes, it indicates that some old genes are still evolving and that provide materials for human evolution through recombination. HI genes may point out the status and future of human evolution as new genes and new functions are generated. We found that tissue-specific genes such as brain and blood specific genes were overrepresented in HI genes. Thus, human HI genes may also be important for human intelligence and the immune system.

### The by-products of meiotic recombination

The purpose of meiotic recombination is not aim to cause diseases, but rather to repair DNA break points. However many disease-associated genes are enriched in HI genes. We first show that Mendelian heritable diseases may be indirect by-products of recombination events during evolution. We also find that chromosomal rearrangement associated diseases are highly associated with hotspot regions. Since many observed chromosomal rearrangement associated diseases are somatic cancers, which are mainly caused by mitotic recombination, it indicates that meiotic recombination may be safe but hotspot sequences may be susceptible to diseases. We also find that hotspot-associated motifs are also enriched in genes involved in chromosomal rearrangement associated diseases. We provide repeat elements that may both promote recombination hotspots and chromosomal rearrangement associated diseases.

## Methods

### Definition of HI gene and dataset collection

We obtained the fine-scale recombination data from decode (released on 2010-10-28, based on human genome assembly NCBI36/hg18) [5]. Recombination hotspot was defined as having a standardized recombination rate (SRR) greater than 10. The standardized sex-averaged recombination maps were used, and 4,008 hotspots in 10 kilo bases resolution were extracted. The sex-averaged recombination maps only included hotspots on the autosomal chromosomes. We used “Intersect” tool in Galaxy [33] to calculate the overlap between HI gene regions and hotspot regions (that is why we named “Hotspot Intersected” gene as “HI gene”). The default coverage parameter of “Intersect” is at least 1 base. We also randomly extracted 4008 middle spots (0.1 < SRR < 1) and 4008 cold spots (SRR = 0). Correspondingly we defined MI gene (middle spot intersected gene) and CI gene (cold spot intersected gene). The RefSeq genes were used as the background reference gene. We first downloaded the RefSeq genes track from UCSC (updated on 2011-07-25) [34,35]. We only retained protein-coding genes that located in the known autosomal chromosomes and named the dataset as hg18rpa. In total, the hg18rpa dataset contained 30,320 mRNA transcripts and 18,166 non-redundant genes (counting official gene symbols) in total. For the comparative analysis of evolution and diseases association, we collected several gene datasets from public databases and the literature (Table 2 Additional file 3). All gene datasets used in this study were mapped to hg18rpa.

**Table 2:** Summary of gene datasets

**Dataset**	**Description**	**Updated**	**Genes**	**Reference**
hg18rpa	Protein-coding genes on autosomal chromosomes (based on human genome hg18 and RefSeq genes)	2011	18,166	[35]
HI	Recombination hotspots intersected genes	2010	1,156	-
MI	Recombination middle spots intersected genes	2010	1,481	-
CI	Recombination cold spots intersected genes	2010	1,594	-
HK	Housekeeping genes	2011	1,974	[36]
SPD	Genes encoding secreted proteins	2010	1,667	[37]
TiGER	Tissue-specific genes	2008	4,710	[38]
DGD	Duplicated genes	2012	4,393	[16]
AGE	Genes classified by different evolutionary age	2011	16,418	[17]
OMIM	Mendelian disorder associated genes in human	2012	2,624	[39]
MD	Mendelian Disease Genes (at least one mutation in the particular gene is causative of the disease)	2011	1,629	[21]
CGC	Genes with mutations have been causally implicated in cancer	2011	424	[23]
TICdb	Reciprocal translocation associated genes in human tumours	2008	240	[40]
dbCRID	Chromosomal rearrangement associated genes in human diseases	2010	401	[22]
InteCR	Integrated chromosomal rearrangement associated genes in human diseases (Combined of dbCRID, TICdb and CGC genes)	2012	614	-

Summary of gene datasets used in this study. All datasets are mapped to hg18rpa. Gene numbers are counted according to non-redundant official gene symbols. Note that HI, MI and CI genes are defined in this study as mentioned above. Detailed gene lists are given in Additional file 3.

### Functional annotation of HI genes

DAVID (The Database for Annotation, Visualization and Integrated Discovery, version 6.7) provided a comprehensive set of online functional annotation tools used to describe the biological meaning behind a large list of genes [41]. RefSeq mRNA identifies of HI genes were submitted to DAVID to perform GO (Gene Ontology) enrichment analysis. The hg18rpa genes were set as the background for comparison. The lowest fold enrichment value was set to 1.5 and the false discovery rate (FDR) was set to 0.05. A total of 2,054 mRNA identifies of HI genes were mapped to 1,142 DAVID genes; and 30,320 mRNA identifies of hg18rpa genes were mapped to 17,626 DAVID genes. There are 833 DAVID HI genes observed in GO-CC (cellular component). Corresponding numbers for GO-MF (molecular function) and GO-BP (biological process) are 802 and 839.

### Evolutionary analysis

For all enrichment analysis in this part, hg18rpa genes were set as background and statistical values were calculated by Fisher’s exact test. For comparison of evolutionary rates, gene datasets were first mapped to ensemble proteins. The dN, dS, and dN/dS values for human-mouse orthologs were directly obtained from Ensemble through BioMart [42]. For the evolutionary comparison of HK genes, TiGER genes, and SPD genes, the common genes with HI genes were excluded in all paired datasets respectively. For example, while comparing the evolutionary rates of HK genes with HI genes, the common genes between HK genes and HI genes were removed to obtain a better representation of the unique features of each dataset. And for comparison of HI genes, MI genes and CI genes, only unique genes in each dataset were retained. The Wilcoxon rank sum non-parametric test was applied for statistical comparison [43]. Duplicated genes were downloaded from the duplicated genes database (DGD) [16]. The DGD genes are co-located to the same chromosome and are highly similar in sequences. Thus, DGD genes are groups of tandem repeated genes. The enrichment comparisons of duplicated genes were also performed by Fisher’s exact test. The non-redundant mRNA transcripts were counted to reflect the feature of gene duplication.

### Disease association analysis

For calculation of the enrichments of disease-associated genes in HI genes, hg18rpa genes were set as background and statistical values were calculated by Fisher’s exact test. A total of 114 fragile sites that located in autosomal chromosomes were extracted from a published dataset and mapped to the hg18 genome [31]. The resolutions of fragile site regions were range from 1E + 3 to1E + 4 kb. The InteCR dataset integrated chromosomal rearrangement associated disease genes in dbCRID, TICdb and CGC genes (only included genes involved in amplification, large deletion, gene conversion, and translocation). A total of 614 InteCR genes and 618 gene regions were obtained. The enrichments of hotspot motifs in InteCR gene regions, HI gene regions, and HI-InteCR regions were calculated by Fisher’s exact test (the hg18rpa gene regions were set as the background). Repeatmasker data, downloaded from UCSC, was used for the analysis of repeated sequences [44]. The fisher’s exact test was used to evaluate the enrichments of repeat elements in datasets.

## Abbreviations

FE: Fold enrichment; P: p value.

## Competing interests

The authors declare that they have no competing interests.

## Authors’ contributions

JS and XG conceived of the project. TZ, ZH and ZZ performed analysis. All authors read and approved the final manuscript.

## Supplementary Material

Additional file 1 Additional and detailed results. Click here for file

Additional file 2 Integrated chromosomal maps of fragile sites, hotspots, HI genes, MD genes and InteCR genes for all autosomal chromosomes. Click here for file

Additional file 3 All gene datasets used in this study. Click here for file
