# Supplementary material for: Genome-wide analysis of human hotspot intersected genes highlights the roles of meiotic recombination in evolution and disease
Source: BMC Genomics. 2013 Jan 31;14:67. doi: 10.1186/1471-2164-14-67 (PMC3620679; doi:10.1186/1471-2164-14-67)
Supplement: Additional file 1 — Additional and detailed results. [file 1471-2164-14-67-S1.docx]

**Additional and detailed results**

## Functional annotation of HI genes

**Table S1** **Enriched GO terms of HI genes**

| **Category** | **Accession** | **Description** | **Count** | **Fold Enrichment** | **FDR** |
| --- | --- | --- | --- | --- | --- |
| CC | GO:0005604 | basement membrane | 18 | 3.5 | 1.2E-02 |
| CC | GO:0044420 | extracellular matrix part | 22 | 2.9 | 2.2E-02 |
| CC | GO:0034703 | cation channel complex | 24 | 2.7 | 3.0E-02 |
| CC | GO:0044456 | synapse part | 41 | 2.5 | 1.6E-04 |
| CC | GO:0045202 | synapse | 58 | 2.5 | 4.0E-07 |
| CC | GO:0034702 | ion channel complex | 32 | 2.4 | 1.5E-02 |
| CC | GO:0005578 | proteinaceous extracellular matrix | 44 | 2.1 | 1.0E-02 |
| CC | GO:0031012 | extracellular matrix | 47 | 2.0 | 6.2E-03 |
| CC | GO:0043005 | neuron projection | 46 | 2.0 | 1.9E-02 |
| CC | GO:0030054 | cell junction | 65 | 1.9 | 1.6E-03 |
| CC | GO:0044459 | plasma membrane part | 230 | 1.6 | 3.6E-11 |
| CC | GO:0044421 | extracellular region part | 101 | 1.6 | 6.2E-03 |
| MF | GO:0019992 | diacylglycerol binding | 16 | 3.7 | 2.7E-02 |
| MF | GO:0022836 | gated channel activity | 49 | 2.5 | 4.6E-06 |
| MF | GO:0005244 | voltage-gated ion channel activity | 31 | 2.5 | 6.4E-03 |
| MF | GO:0022832 | voltage-gated channel activity | 31 | 2.5 | 6.4E-03 |
| MF | GO:0022838 | substrate specific channel activity | 61 | 2.4 | 1.9E-07 |
| MF | GO:0046873 | metal ion transmembrane transporter activity | 51 | 2.4 | 9.2E-06 |
| MF | GO:0005261 | cation channel activity | 43 | 2.4 | 1.8E-04 |
| MF | GO:0005216 | ion channel activity | 58 | 2.4 | 1.3E-06 |
| MF | GO:0015267 | channel activity | 61 | 2.4 | 7.8E-07 |
| MF | GO:0022803 | passive transmembrane transporter activity | 61 | 2.4 | 8.6E-07 |
| MF | GO:0031420 | alkali metal ion binding | 34 | 2.3 | 1.8E-02 |
| MF | GO:0005509 | calcium ion binding | 125 | 2.1 | 1.8E-13 |
| MF | GO:0030695 | GTPase regulator activity | 49 | 1.9 | 1.8E-02 |
| MF | GO:0060589 | nucleoside-triphosphatase regulator activity | 49 | 1.9 | 3.3E-02 |
| BP | GO:0007411 | axon guidance | 26 | 3.9 | 1.2E-05 |
| BP | GO:0048667 | cell morphogenesis involved in neuron differentiation | 41 | 3.1 | 3.3E-07 |
| BP | GO:0048812 | neuron projection morphogenesis | 40 | 3.0 | 1.6E-06 |
| BP | GO:0007409 | axonogenesis | 36 | 3.0 | 2.0E-05 |
| BP | GO:0000904 | cell morphogenesis involved in differentiation | 44 | 2.9 | 1.0E-06 |
| BP | GO:0031175 | neuron projection development | 44 | 2.7 | 4.0E-06 |
| BP | GO:0043062 | extracellular structure organization | 27 | 2.6 | 1.9E-02 |
| BP | GO:0048858 | cell projection morphogenesis | 40 | 2.6 | 1.1E-04 |
| BP | GO:0032990 | cell part morphogenesis | 40 | 2.5 | 3.9E-04 |
| BP | GO:0048666 | neuron development | 53 | 2.5 | 4.2E-06 |
| BP | GO:0000902 | cell morphogenesis | 52 | 2.3 | 4.9E-05 |
| BP | GO:0030182 | neuron differentiation | 62 | 2.2 | 1.0E-05 |
| BP | GO:0016337 | cell-cell adhesion | 39 | 2.2 | 1.0E-02 |
| BP | GO:0032989 | cellular component morphogenesis | 54 | 2.1 | 3.1E-04 |
| BP | GO:0030030 | cell projection organization | 50 | 2.1 | 9.4E-04 |
| BP | GO:0030001 | metal ion transport | 63 | 2.1 | 3.5E-05 |
| BP | GO:0015672 | monovalent inorganic cation transport | 42 | 2.1 | 2.4E-02 |
| BP | GO:0006928 | cell motion | 61 | 2.0 | 6.3E-04 |
| BP | GO:0007155 | cell adhesion | 87 | 2.0 | 3.0E-06 |
| BP | GO:0022610 | biological adhesion | 87 | 2.0 | 3.1E-06 |
| BP | GO:0006812 | cation transport | 67 | 1.9 | 8.3E-04 |
| BP | GO:0006811 | ion transport | 88 | 1.8 | 7.8E-05 |

Enrichments of HI genes are calculated by DAVID bioinformatics compared to hg18rpa genes. CC, MF and BP represents cellular component, molecular function and biological process respectively in Gene Ontology. FDR stands for False Discovery Rate, DAVID calculated FDR based on the P values of Fisher’s exact test.

## HI genes and human evolution

**Table S2 Comparison of evolutionary rates**

| **Gene dataset** | **Evolutionary rate** | **Median values** | **P value** |
| --- | --- | --- | --- |
| HK | dN | 0.033 (0.056) | <0.0001 |
| HK | dS | 0.497 (0.605) | <0.0001 |
| HK | dN/dS | 0.068 (0.088) | <0.0001 |
| TiGER | dN | 0.094 (0.051) | <0.0001 |
| TiGER | dS | 0.651 (0.590) | <0.0001 |
| TiGER | dN/dS | 0.140 (0.084) | <0.0001 |
| SPD | dN | 0.136 (0.048) | <0.0001 |
| SPD | dS | 0.672 (0.597) | <0.0001 |
| SPD | dN/dS | 0.196 (0.082) | <0.0001 |
| MI | dN | 0.054 (0.067) | 0.0003 |
| MI | dS | 0.561 (0.645) | <0.0001 |
| MI | dN/dS | 0.101 (0.102) | 0.83 |
| CI | dN | 0.051 (0.067) | <0.0001 |
| CI | dS | 0.517 (0.645) | <0.0001 |
| CI | dN/dS | 0.101 (0.102) | 0.008 |

The evolutionary rates including dN, dS and dN/dS values of each gene dataset are compared to HI genes. For HK genes, TiGER genes and SPD genes, the same genes with HI genes are excluded in both datasets respectively. And for comparison of HI genes, MI genes and CI genes, only unique genes in each dataset are kept. The corresponding median values of HI genes are showed in brackets.

**Table S3** **Standard Deviations of evolutionary rates**

| **Gene dataset** | **Evolutionary rate** | **Standard Deviations** | |
| --- | --- | --- | --- |
| HK | dN | | 0.096 (0.141) |
| HK | dS | | 1.964 (0.622) |
| HK | dN/dS | | 0.128 (0.167) |
| TiGER | dN | | 0.269 (0.111) |
| TiGER | dS | | 2.313 (0.243) |
| TiGER | dN/dS | | 0.200 (0.144) |
| SPD | dN | | 0.348 (0.094) |
| SPD | dS | | 1.828 (0.252) |
| SPD | dN/dS | | 1.785 (0.112) |
| MI | dN | | 0.130 (0.155) |
| MI | dS | | 1.224 (0.714) |
| MI | dN/dS | | 0.158 (0.182) |
| CI | dN | | 0.224 (0.155) |
| CI | dS | | 0.204 (0.714) |
| CI | dN/dS | | 0.164 (0.182) |

Standard Deviations for the comparison of evolutionary rates are listed. The corresponding values of HI genes are showed in brackets.

**Table S4 Comparison of duplicated genes**

| **Dataset** | **DGD count** | **Total count** | **Proportion** | **P value** |
| --- | --- | --- | --- | --- |
| HI | 413 | 2,054 | 20.11% | - |
| HK | 349 | 3,522 | 9.91% | <0.0001 |
| TiGER | 1,491 | 5,821 | 25.61% | <0.0001 |
| SPD | 988 | 2,972 | 33.24% | <0.0001 |
| MI | 512 | 2,706 | 18.92% | >0.05 |
| CI | 386 | 2,790 | 13.84% | <0.0001 |
| hg18rpa | 6,655 | 29,988 | 22.19% | >0.05 |

Counts and proportions of DGD transcripts in different datasets are shown. The proportions of HK transcripts, TiGER transcripts, SPD transcripts, MI transcripts, CI transcripts and hg18rpa transcripts are compared to HI transcripts.

## HI genes and human diseases Table S5 Enrichments of Hotspot motifs

| **Motif** | **Gene regions** | **Hit motif regions** | **Total regions** | **Fold enrichmnt** | **P value** |
| --- | --- | --- | --- | --- | --- |
| CCTCCCT | HI | 1,121 | 1,136 | 1.1 | 1.17E-01 |
| CCTCCCT | InteCR | 599 | 618 | 1.1 | 3.97E-01 |
| CCTCCCT | HI-InteCR | 92 | 92 | 1.1 | 6.05E-01 |
| CCTCCCT | hg18rpa | 15,553 | 16,885 | - | - |
| CCCCACCCC | HI | 1,034 | 1,136 | 1.3 | 1.53E-09 |
| CCCCACCCC | InteCR | 542 | 618 | 1.3 | 1.10E-04 |
| CCCCACCCC | HI-InteCR | 92 | 92 | 1.4 | 1.59E-02 |
| CCCCACCCC | hg18rpa | 11,721 | 16,885 | - | - |
| CCNCCNTNNCCNC | HI | 1,093 | 1,136 | 1.1 | 2.40E-03 |
| CCNCCNTNNCCNC | InteCR | 578 | 618 | 1.1 | 7.60E-02 |
| CCNCCNTNNCCNC | HI-InteCR | 92 | 92 | 1.2 | 2.66E-01 |
| CCNCCNTNNCCNC | hg18rpa | 14,216 | 16,885 | - | - |

Number of regions and fold enrichment for each motif in HI gene regions, InteCR gene regions and HI-InteCR gene regions are listed. Enrichments and p values are calculated by comparing each gene regions to hg18rpa gene regions.

**Table S6 Enrichments of repeat elements in hotspot regions**

| **Repeat element** | **Repeat class** | **Repeat count** | **Fold enrichment** | **P value** |
| --- | --- | --- | --- | --- |
| (TGG)n | Simple_repeat | 64 | 3.5 | 2.20E-16 |
| CT-rich | Low_complexity | 563 | 1.7 | 2.20E-16 |
| MLT1K | LTR | 439 | 1.6 | 2.20E-16 |
| MIRm | SINE | 722 | 1.6 | 2.20E-16 |
| MIRb | SINE | 5,873 | 1.5 | 2.20E-16 |
| MIR3 | SINE | 1,449 | 1.4 | 2.20E-16 |
| MIR | SINE | 4,124 | 1.4 | 2.20E-16 |
| (TG)n | Simple_repeat | 1,028 | 1.3 | 2.20E-16 |
| L2 | LINE | 7,471 | 1.3 | 2.20E-16 |
| MLT1C | LTR | 418 | 1.5 | 4.54E-16 |
| MER5A1 | DNA | 326 | 1.6 | 1.62E-13 |
| MER5B | DNA | 497 | 1.4 | 1.91E-13 |
| MER5A | DNA | 675 | 1.3 | 4.91E-13 |
| (TCCA)n | Simple_repeat | 102 | 2.0 | 3.74E-10 |
| LTR16C | LTR | 203 | 1.6 | 6.28E-10 |
| Charlie8 | DNA | 181 | 1.6 | 2.58E-09 |
| (CCCCCA)n | Simple_repeat | 23 | 4.8 | 3.13E-09 |
| (ATG)n | Simple_repeat | 69 | 2.2 | 4.24E-09 |
| GA-rich | Low_complexity | 449 | 1.3 | 5.75E-09 |
| MER113 | DNA | 116 | 1.8 | 1.95E-08 |
| MER45 | DNA | 33 | 3.1 | 4.61E-08 |
| MER91A | DNA | 90 | 1.9 | 5.49E-08 |
| (CA)n | Simple_repeat | 907 | 1.2 | 2.86E-07 |
| MER96 | DNA | 43 | 2.4 | 6.79E-07 |
| MER53 | DNA | 129 | 1.6 | 7.94E-07 |
| (TGAA)n | Simple_repeat | 112 | 1.7 | 9.82E-07 |
| (CCCCAA)n | Simple_repeat | 11 | 7.0 | 1.03E-06 |
| (TTCC)n | Simple_repeat | 84 | 1.8 | 1.21E-06 |
| MER103 | DNA | 149 | 1.5 | 1.21E-06 |
| MER63A | DNA | 73 | 1.9 | 1.30E-06 |
| MER66B-int | LTR | 12 | 6.0 | 1.33E-06 |
| THE1B | LTR | 397 | 1.3 | 1.67E-06 |
| MER63 | DNA | 85 | 1.7 | 3.84E-06 |
| MLT1H | LTR | 193 | 1.4 | 6.14E-06 |
| (TCCC)n | Simple_repeat | 44 | 2.1 | 7.41E-06 |
| LTR33 | LTR | 188 | 1.4 | 1.36E-05 |
| L3 | LINE | 765 | 1.2 | 1.38E-05 |
| LTR32 | LTR | 40 | 2.1 | 2.07E-05 |
| (GAGTG)n | Simple_repeat | 10 | 5.9 | 2.24E-05 |
| MER81 | DNA | 85 | 1.6 | 2.45E-05 |
| (T)n | Simple_repeat | 251 | 1.3 | 4.68E-05 |
| Tigger5 | DNA | 75 | 1.7 | 5.34E-05 |
| MER20 | DNA | 291 | 1.3 | 6.14E-05 |
| ERVL-E | LTR | 187 | 1.4 | 6.54E-05 |
| (CAT)n | Simple_repeat | 56 | 1.8 | 6.58E-05 |
| Kanga2_a | DNA | 42 | 2.0 | 7.48E-05 |
| MER66B | LTR | 40 | 2.0 | 7.70E-05 |
| MLT1I | LTR | 214 | 1.3 | 1.05E-04 |
| MLT1B | LTR | 309 | 1.3 | 1.17E-04 |
| (CTACT)n | Simple_repeat | 5 | 11.1 | 1.35E-04 |
| Charlie4a | DNA | 55 | 1.7 | 1.53E-04 |
| (CTATT)n | Simple_repeat | 11 | 4.0 | 1.73E-04 |
| MER94 | DNA | 100 | 1.5 | 1.76E-04 |
| (TAGG)n | Simple_repeat | 11 | 3.7 | 2.66E-04 |
| MER91 | DNA | 23 | 2.4 | 2.90E-04 |
| MER58 | DNA | 59 | 1.6 | 5.79E-04 |
| L3b | LINE | 146 | 1.4 | 5.84E-04 |
| (TTCA)n | Simple_repeat | 96 | 1.5 | 6.59E-04 |
| MER45C | DNA | 30 | 2.0 | 7.14E-04 |
| MER68-int | LTR | 11 | 3.3 | 7.24E-04 |
| MER20B | DNA | 85 | 1.5 | 7.31E-04 |
| MER121 | Unknown | 27 | 2.0 | 8.36E-04 |
| polypurine | Low_complexity | 50 | 1.7 | 8.65E-04 |
| (GA)n | Simple_repeat | 167 | 1.3 | 9.80E-04 |
| MER102b | DNA | 83 | 1.5 | 1.06E-03 |
| MLT1A0 | LTR | 342 | 1.2 | 1.15E-03 |
| PABL_A-int | LTR | 11 | 3.2 | 1.29E-03 |
| MLT1J2 | LTR | 149 | 1.3 | 1.30E-03 |
| MER45A | DNA | 69 | 1.5 | 1.33E-03 |
| MSR1 | Satellite | 13 | 2.8 | 1.44E-03 |
| MLT1L | LTR | 207 | 1.3 | 1.46E-03 |
| MER93B-int | LTR | 6 | 4.8 | 1.65E-03 |
| MER117 | DNA | 90 | 1.4 | 1.98E-03 |
| (AGTAG)n | Simple_repeat | 3 | 11.7 | 2.21E-03 |
| L4 | LINE | 280 | 1.2 | 2.37E-03 |
| (GGCTG)n | Simple_repeat | 10 | 3.1 | 2.42E-03 |
| MER58A | DNA | 214 | 1.2 | 2.45E-03 |
| MER3 | DNA | 195 | 1.3 | 2.48E-03 |
| MER115 | DNA | 54 | 1.5 | 2.74E-03 |
| (CACCAT)n | Simple_repeat | 19 | 2.1 | 3.98E-03 |
| (CATAT)n | Simple_repeat | 7 | 3.5 | 4.05E-03 |
| (TTCCC)n | Simple_repeat | 12 | 2.5 | 4.28E-03 |
| LTR59-int | LTR | 2 | 21.0 | 4.34E-03 |
| (CAGAGA)n | Simple_repeat | 28 | 1.8 | 4.97E-03 |
| tRNA-Cys-TGY | tRNA | 7 | 3.5 | 5.33E-03 |
| MER96B | DNA | 46 | 1.5 | 5.49E-03 |
| (GGAA)n | Simple_repeat | 66 | 1.4 | 6.16E-03 |
| MLT1D | LTR | 328 | 1.2 | 6.18E-03 |
| (TC)n | Simple_repeat | 161 | 1.2 | 6.94E-03 |
| HUERS-P2 | LTR | 9 | 2.6 | 7.62E-03 |
| LTR57 | LTR | 9 | 2.6 | 8.03E-03 |
| MER106B | DNA | 22 | 1.8 | 8.29E-03 |
| PABL_B-int | LTR | 7 | 3.3 | 8.47E-03 |
| MLT1J | LTR | 264 | 1.2 | 8.54E-03 |
| MER4E-int | LTR | 6 | 3.6 | 8.97E-03 |
| MER65B-int | LTR | 4 | 5.1 | 9.40E-03 |
| (TTG)n | Simple_repeat | 104 | 1.3 | 9.41E-03 |
| MER90a | LTR | 27 | 1.7 | 1.20E-02 |
| (CTTAA)n | Simple_repeat | 2 | 11.1 | 1.30E-02 |
| MSTC | LTR | 61 | 1.4 | 1.49E-02 |
| (TTGTG)n | Simple_repeat | 4 | 4.7 | 1.56E-02 |
| MLT1A1 | LTR | 117 | 1.3 | 1.61E-02 |
| tRNA-Ile-ATA | tRNA | 4 | 4.5 | 1.65E-02 |
| LTR38 | LTR | 10 | 2.2 | 1.68E-02 |
| MER58B | DNA | 122 | 1.2 | 1.93E-02 |
| THE1A | LTR | 76 | 1.3 | 2.03E-02 |
| (CCTAT)n | Simple_repeat | 3 | 4.8 | 2.10E-02 |
| MER33 | DNA | 136 | 1.2 | 2.19E-02 |
| MLT1H2 | LTR | 78 | 1.3 | 2.24E-02 |
| LTR16A | LTR | 120 | 1.2 | 2.46E-02 |
| LTR29 | LTR | 16 | 1.9 | 2.49E-02 |
| tRNA-Gln-CAG | tRNA | 4 | 3.4 | 2.50E-02 |
| LTR54B | LTR | 20 | 1.7 | 2.73E-02 |
| MER91C | DNA | 27 | 1.6 | 3.08E-02 |
| AluJb_short_ | SINE | 5 | 2.6 | 3.40E-02 |
| (ATGGTG)n | Simple_repeat | 17 | 1.8 | 3.45E-02 |
| (CAACC)n | Simple_repeat | 2 | 6.3 | 3.46E-02 |
| (TTTC)n | Simple_repeat | 60 | 1.3 | 3.69E-02 |
| (GGGA)n | Simple_repeat | 29 | 1.5 | 3.78E-02 |
| MLT2C2 | LTR | 29 | 1.5 | 3.79E-02 |
| ORSL | DNA | 26 | 1.5 | 3.82E-02 |
| LTR9B | LTR | 17 | 1.7 | 3.85E-02 |
| (TCTG)n | Simple_repeat | 13 | 1.9 | 3.94E-02 |
| MER44B | DNA | 27 | 1.5 | 4.09E-02 |
| MER61-int | LTR | 6 | 2.6 | 4.10E-02 |
| LTR45B | LTR | 12 | 1.8 | 4.14E-02 |
| (TTCGG)n | Simple_repeat | 1 | 35.7 | 4.35E-02 |
| L1ME4a | LINE | 588 | 1.1 | 4.37E-02 |
| MLT1A | LTR | 150 | 1.2 | 4.39E-02 |
| LTR37A | LTR | 39 | 1.4 | 4.45E-02 |

Number of each type of repeat observed in hotspots and corresponding fold enrichments are listed. Enrichment and p values are calculated by comparing each gene regions to hg18 autosomal genome regions. Data are sorted by p values.

**Table S7 Enrichments of repeat elements in HI-InteCR gene regions**

| **Repeat element** | **Repeat class** | **Repeat count** | **Fold enrichment** | **P value** |
| --- | --- | --- | --- | --- |
| MIRb | SINE | 4,404 | 1.2 | 2.20E-16 |
| MIR | SINE | 2,973 | 1.2 | 3.67E-12 |
| L2 | LINE | 5,138 | 1.1 | 2.75E-09 |
| MIR3 | SINE | 1,218 | 1.2 | 1.75E-08 |
| MLT1K | LTR | 249 | 1.6 | 3.97E-08 |
| MLT1C | LTR | 228 | 1.4 | 5.40E-05 |
| MLT1A0 | LTR | 214 | 1.4 | 1.52E-04 |
| LTR16C | LTR | 67 | 1.8 | 3.20E-04 |
| THE1B | LTR | 225 | 1.3 | 4.22E-04 |
| MLT1J2 | LTR | 85 | 1.7 | 4.83E-04 |
| MER5A | DNA | 526 | 1.2 | 8.51E-04 |
| CT-rich | Low_complexity | 325 | 1.3 | 1.02E-03 |
| MIRm | SINE | 509 | 1.2 | 1.42E-03 |
| (CA)n | Simple_repeat | 771 | 1.1 | 1.70E-03 |
| MLT1I | LTR | 124 | 1.4 | 2.94E-03 |
| (ATG)n | Simple_repeat | 49 | 1.8 | 3.03E-03 |
| L3 | LINE | 693 | 1.1 | 3.07E-03 |
| (TG)n | Simple_repeat | 769 | 1.1 | 3.63E-03 |
| MER5B | DNA | 325 | 1.2 | 5.20E-03 |
| (CACCAT)n | Simple_repeat | 19 | 2.5 | 6.33E-03 |
| LTR16A | LTR | 65 | 1.6 | 6.53E-03 |
| L4 | LINE | 253 | 1.2 | 7.67E-03 |
| MER5A1 | DNA | 196 | 1.3 | 9.34E-03 |
| MER117 | DNA | 97 | 1.4 | 1.09E-02 |
| MLT1D | LTR | 198 | 1.2 | 1.13E-02 |
| (TTCC)n | Simple_repeat | 43 | 1.6 | 1.43E-02 |
| (TGAA)n | Simple_repeat | 90 | 1.4 | 1.53E-02 |
| MER58A | DNA | 203 | 1.2 | 1.63E-02 |
| (CAT)n | Simple_repeat | 49 | 1.6 | 1.87E-02 |
| LTR32 | LTR | 19 | 2.0 | 2.81E-02 |
| MER113 | DNA | 90 | 1.3 | 3.39E-02 |
| MER45A | DNA | 45 | 1.5 | 3.41E-02 |
| LTR33 | LTR | 77 | 1.4 | 3.68E-02 |
| MLT1B | LTR | 163 | 1.2 | 3.80E-02 |
| MLT1H | LTR | 78 | 1.3 | 3.93E-02 |
| MER53 | DNA | 70 | 1.4 | 4.18E-02 |
| MLT1A | LTR | 83 | 1.3 | 4.63E-02 |
| HERVL18 | LTR | 16 | 2.6 | 8.87E-03 |
| MER61A | LTR | 12 | 2.2 | 4.71E-02 |
| MER63D | DNA | 20 | 1.9 | 3.63E-02 |
| LTR16A1 | LTR | 36 | 1.9 | 5.27E-03 |
| LTR7 | LTR | 22 | 1.9 | 2.87E-02 |
| Arthur1 | DNA | 51 | 1.8 | 1.28E-03 |
| MLT2B1 | LTR | 39 | 1.8 | 6.16E-03 |
| L1MA10 | LINE | 69 | 1.8 | 4.24E-04 |
| polypyrimidine | Low_complexity | 37 | 1.6 | 3.18E-02 |
| MLT1G1 | LTR | 53 | 1.4 | 3.43E-02 |
| L1MA6 | LINE | 54 | 1.4 | 3.70E-02 |
| L1MB1 | LINE | 69 | 1.4 | 2.51E-02 |
| MSTA | LTR | 150 | 1.3 | 2.51E-02 |
| (TA)n | Simple_repeat | 767 | 1.2 | 8.29E-05 |

Number of each type of repeat observed in HI-InteCR gene regions and corresponding fold enrichments are listed. Enrichment and p values are calculated by comparing each gene regions to InteCR gene regions. Data are sorted by p values.

**Table S8 Enriched repeat elements in HI-InteCR gene regions**

| **Repeat element** | **Repeat class** | **Repeat count** | **Fold enrichment** | **P value** |
| --- | --- | --- | --- | --- |
| MIRb | SINE | 4404 | 1.2 | 2.20E-16 |
| MIR | SINE | 2973 | 1.2 | 3.67E-12 |
| L2 | LINE | 5138 | 1.1 | 2.75E-09 |
| MIR3 | SINE | 1218 | 1.2 | 1.75E-08 |
| MLT1K | LTR | 249 | 1.6 | 3.97E-08 |
| MLT1C | LTR | 228 | 1.4 | 5.40E-05 |
| MLT1A0 | LTR | 214 | 1.4 | 1.52E-04 |
| LTR16C | LTR | 67 | 1.8 | 3.20E-04 |
| THE1B | LTR | 225 | 1.3 | 4.22E-04 |
| MLT1J2 | LTR | 85 | 1.7 | 4.83E-04 |
| MER5A | DNA | 526 | 1.2 | 8.51E-04 |
| CT-rich | Low_complexity | 325 | 1.3 | 1.02E-03 |
| MIRm | SINE | 509 | 1.2 | 1.42E-03 |
| (CA)n | Simple_repeat | 771 | 1.1 | 1.70E-03 |
| MLT1I | LTR | 124 | 1.4 | 2.94E-03 |
| (ATG)n | Simple_repeat | 49 | 1.8 | 3.03E-03 |
| L3 | LINE | 693 | 1.1 | 3.07E-03 |
| (TG)n | Simple_repeat | 769 | 1.1 | 3.63E-03 |
| MER5B | DNA | 325 | 1.2 | 5.20E-03 |
| (CACCAT)n | Simple_repeat | 19 | 2.5 | 6.33E-03 |
| LTR16A | LTR | 65 | 1.6 | 6.53E-03 |
| L4 | LINE | 253 | 1.2 | 7.67E-03 |
| MER5A1 | DNA | 196 | 1.3 | 9.34E-03 |
| MER117 | DNA | 97 | 1.4 | 1.09E-02 |
| MLT1D | LTR | 198 | 1.2 | 1.13E-02 |
| (TTCC)n | Simple_repeat | 43 | 1.6 | 1.43E-02 |
| (TGAA)n | Simple_repeat | 90 | 1.4 | 1.53E-02 |
| MER58A | DNA | 203 | 1.2 | 1.63E-02 |
| (CAT)n | Simple_repeat | 49 | 1.6 | 1.87E-02 |
| LTR32 | LTR | 19 | 2.0 | 2.81E-02 |
| MER113 | DNA | 90 | 1.3 | 3.39E-02 |
| MER45A | DNA | 45 | 1.5 | 3.41E-02 |
| LTR33 | LTR | 77 | 1.4 | 3.68E-02 |
| MLT1B | LTR | 163 | 1.2 | 3.80E-02 |
| MLT1H | LTR | 78 | 1.3 | 3.93E-02 |
| MER53 | DNA | 70 | 1.4 | 4.18E-02 |
| MLT1A | LTR | 83 | 1.3 | 4.63E-02 |

A total of 37 repeat elements are both enriched in HI-InteCR gene regions and hotspot regions. Repeat count indicates the observed repeat number in HI-InteCR gene regions. Enrichments and p values are calculated by comparing HI-InteCR gene regions to InteCR gene regions. Data are sorted by p value.

## Additional result Table S9 Summary of gene enrichment analysis

| **Gene dataset** | **Hit HI genes** | **Total count** | **Fold enrichment** | **P value** |
| --- | --- | --- | --- | --- |
| SPD genes | 144 | 1,667 | 1.4 | 1.25E-03 |
| TiGER genes | 384 | 4,710 | 1.3 | 6.67E-05 |
| mammary_gland (TiGER tissue) | 12 | 95 | 2.0 | 3.72E-02 |
| tongue (TiGER tissue) | 32 | 306 | 1.6 | 1.10E-02 |
| colon (TiGER tissue) | 20 | 160 | 2.0 | 7.17E-03 |
| eye (TiGER tissue) | 23 | 193 | 1.9 | 8.73E-03 |
| kidney (TiGER tissue) | 33 | 310 | 1.7 | 8.17E-03 |
| lymph_node (TiGER tissue) | 32 | 298 | 1.7 | 9.72E-03 |
| soft_tissue (TiGER tissue) | 17 | 121 | 2.2 | 5.74E-03 |
| spleen (TiGER tissue) | 16 | 120 | 2.1 | 9.83E-03 |
| blood (TiGER tissue) | 39 | 335 | 1.8 | 9.38E-04 |
| brain (TiGER tissue) | 34 | 203 | 2.6 | 3.11E-06 |
| bladder (TiGER tissue) | 5 | 162 | 0.5 | 1.36E-01 |
| bone (TiGER tissue) | 9 | 96 | 1.5 | 2.97E-01 |
| bone_marrow (TiGER tissue) | 15 | 230 | 1.0 | 8.92E-01 |
| cervix (TiGER tissue) | 6 | 185 | 0.5 | 1.22E-01 |
| heart (TiGER tissue) | 13 | 195 | 1.0 | 8.83E-01 |
| larynx (TiGER tissue) | 19 | 241 | 1.2 | 3.57E-01 |
| liver (TiGER tissue) | 13 | 278 | 0.7 | 3.19E-01 |
| lung (TiGER tissue) | 8 | 95 | 1.3 | 4.04E-01 |
| muscle (TiGER tissue) | 11 | 218 | 0.8 | 5.74E-01 |
| ovary (TiGER tissue) | 13 | 116 | 1.8 | 6.08E-02 |
| pancreas (TiGER tissue) | 9 | 145 | 1.0 | 1.00E+00 |
| peripheral_nervous_system (TiGER tissue) | 5 | 85 | 0.9 | 1.00E+00 |
| placenta (TiGER tissue) | 18 | 220 | 1.3 | 3.34E-01 |
| prostate (TiGER tissue) | 10 | 115 | 1.4 | 3.41E-01 |
| skin (TiGER tissue) | 3 | 106 | 0.4 | 2.19E-01 |
| small_intestine (TiGER tissue) | 6 | 96 | 1.0 | 1.00E+00 |
| stomach (TiGER tissue) | 11 | 170 | 1.0 | 8.75E-01 |
| testis (TiGER tissue) | 24 | 550 | 0.7 | 7.29E-02 |
| thymus (TiGER tissue) | 5 | 72 | 1.1 | 8.08E-01 |
| uterus (TiGER tissue) | 5 | 38 | 2.1 | 1.83E-01 |
| HK genes | 29 | 1,974 | 0.2 | 2.20E-16 |
| OMIM | 219 | 2,624 | 1.3 | 5.41E-04 |
| MD | 141 | 1,629 | 1.4 | 1.32E-03 |
| CGC | 45 | 424 | 1.7 | 2.29E-03 |
| TICdb | 29 | 240 | 1.9 | 2.69E-03 |
| CGC | 45 | 424 | 1.7 | 2.29E-03 |
| amplification (CGC mutation type) | 0 | 16 | 0.0 | 6.20E-01 |
| large deletion (CGC mutation type) | 1 | 32 | 0.5 | 7.21E-01 |
| frameshift (CGC mutation type) | 2 | 86 | 0.4 | 1.76E-01 |
| Gene Conversion (CGC mutation type) | 0 | 1 | 0.0 | 1.00E+00 |
| Missense (CGC mutation type) | 4 | 128 | 0.5 | 1.95E-01 |
| nonsense (CGC mutation type) | 1 | 82 | 0.2 | 6.33E-02 |
| other Mutation Type (CGC mutation type) | 1 | 23 | 0.7 | 1.00E+00 |
| splice site (CGC mutation type) | 2 | 52 | 0.6 | 7.71E-01 |
| translocation (CGC mutation type) | 40 | 282 | 2.2 | 1.70E-05 |
| epithelial (CGC tissue type) | 12 | 146 | 1.3 | 3.97E-01 |
| leukaemia/lymphoma (CGC tissue type) | 29 | 241 | 1.9 | 2.76E-03 |
| mesenchymal (CGC tissue type) | 13 | 80 | 2.6 | 3.72E-03 |
| other tissue (CGC tissue type) | 2 | 45 | 0.7 | 1.00E+00 |
| Germline (CGC cell type) | 2 | 73 | 0.4 | 3.27E-01 |
| Somatic (CGC cell type) | 44 | 385 | 1.8 | 6.73E-04 |
| dbCRID | 73 | 401 | 2.9 | 5.33E-13 |
| Reciprocal Translocation (dbCRID subclass) | 56 | 285 | 3.1 | 1.57E-11 |
| Deletion (dbCRID subclass) | 19 | 124 | 2.4 | 1.12E-03 |
| Duplication (dbCRID subclass) | 2 | 11 | 2.9 | 1.81E-01 |
| Inversion (dbCRID subclass) | 8 | 35 | 3.6 | 3.67E-03 |
| Insertion (dbCRID subclass) | 3 | 12 | 3.9 | 5.69E-02 |
| Translocation (dbCRID subclass) | 1 | 3 | 5.2 | 2.19E-01 |
| Ring Chromosome (dbCRID subclass) | 0 | 2 | 0.0 | 1.00E+00 |
| InteCR | 92 | 614 | 2.4 | 1.83E-11 |

All enrichment results for evolutionary and diseases association analyses are listed. Enrichment and p values are calculated by comparing each gene dataset to hg18rpa.
