# Supplementary material for: Genome-wide analysis of human hotspot intersected genes highlights the roles of meiotic recombination in evolution and disease
Source: BMC Genomics. 2013 Jan 31;14:67. doi: 10.1186/1471-2164-14-67 (PMC3620679; doi:10.1186/1471-2164-14-67)
Supplement: Additional file 2 — Integrated chromosomal maps of fragile sites, hotspots, HI genes, MD genes and InteCR genes for all autosomal chromosomes. [file 1471-2164-14-67-S2.pdf]

Graphs show the integrated chromosomal maps for hotspots, fragile sites, HI genes and disease genes. Chromosomal positions and bands are based on human genome hg18 obtained from UCSC.
The first rentangle stands for chromosomal ideograms.
Scheme for bands:

color	note
	gneg - Giemsa negative bands
	gpos25 - Giemsa positive bands
	gpos50 - Giemsa positive bands
	gpos75 - Giemsa positive bands
	gpos100 - Giemsa positive bands
	acen - centromeric regions
	gvar - variable length heterochromatic regions
	stalk - tightly constricted regions on the short arms of the acrocentric chromosomes

*The Giemsa positive bands have further been subdivided into gpos25, gpos50, gpos75, and gpos100 with the higher number indicating a darker stain.

Each orange region in the second rectangle stands for a fragile site location.

Each red line in the third rectangle stands for a hotspot(10k bp).

The gray lines in the rightmost part of the graph stand for hg18rpa genes. The length of each line reflects the relative length of a gene. HI genes are colored red and annotated with '-' or '+' based on gene types in the left.
Scheme for gene types:

Color	Gene type
red	MD genes
purple	InteCR genes

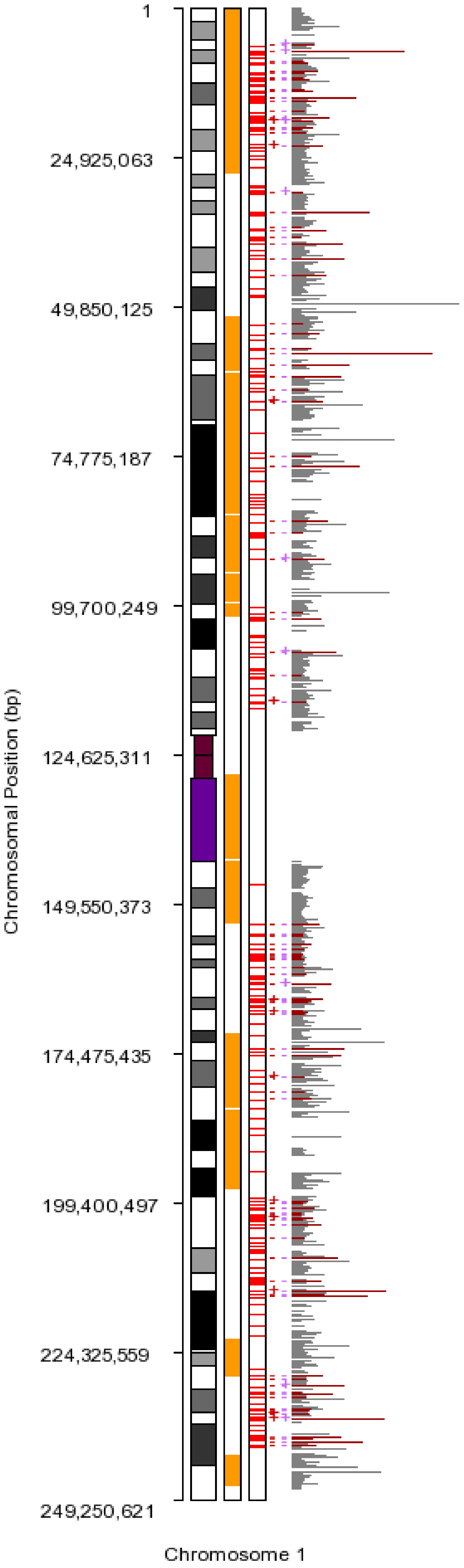
1
24,925,063
49,850,125
74,775,187
99,700,249
124,625,311
149,550,373
174,475,435
199,400,497
224,325,559
249,250,621
Chromosomal Position (bp)
Chromosome 1

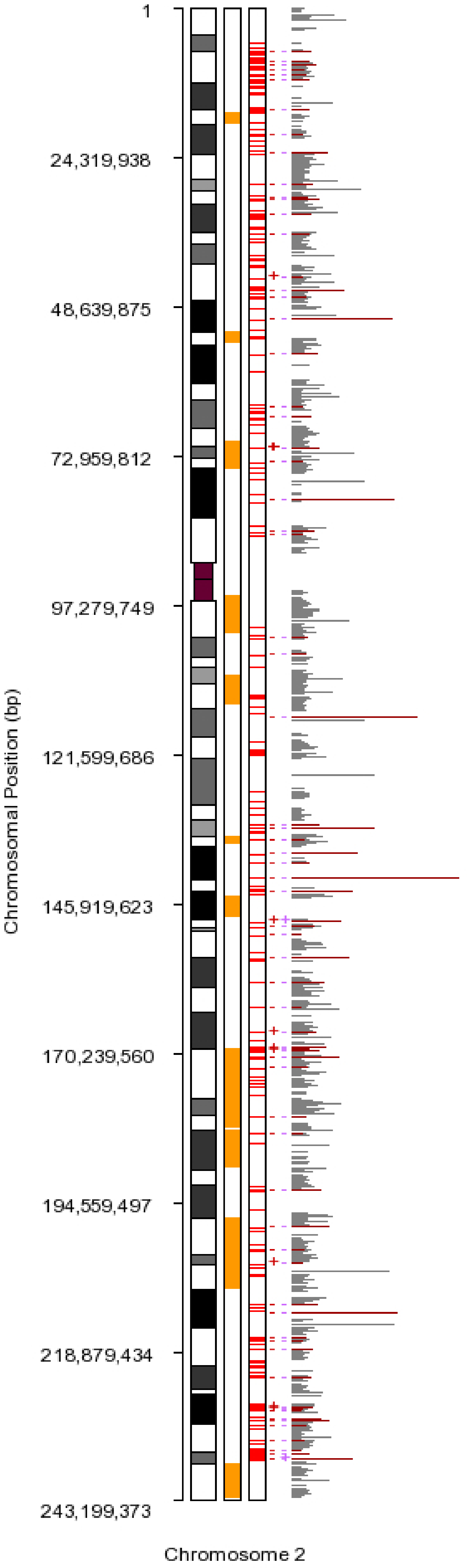
1
24,319,938
48,639,875
72,959,812
97,279,749
121,599,686
145,919,623
170,239,560
194,559,497
218,879,434
243,199,373
Chromosomal Position (bp)
Chromosome 2

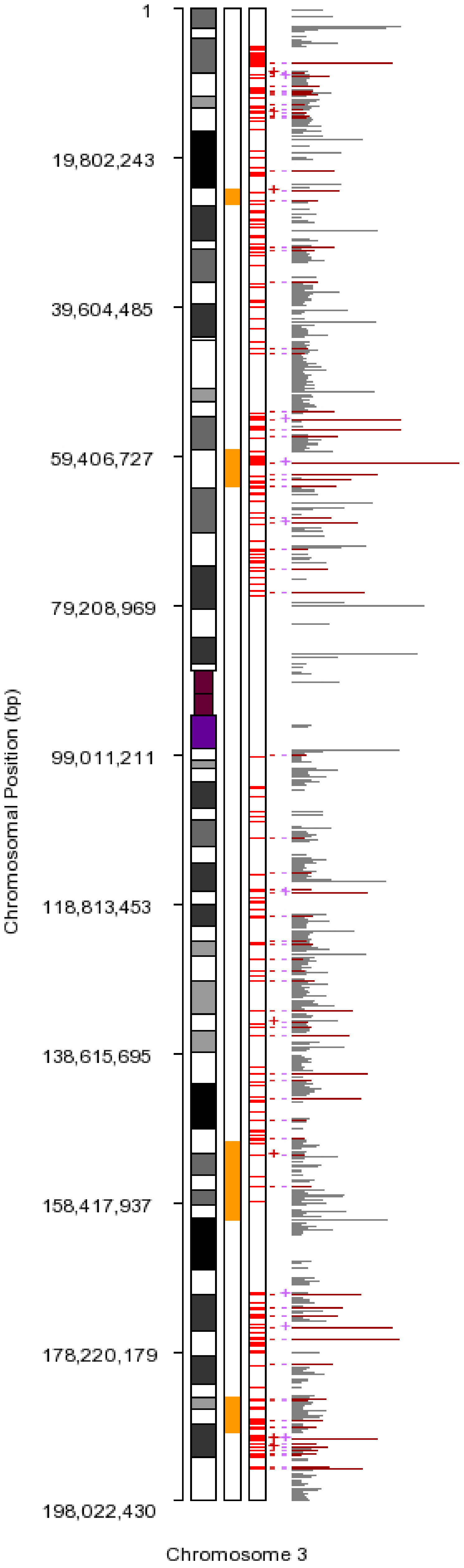
1
19,802,243
39,604,485
59,406,727
79,208,969
99,011,211
118,813,453
138,615,695
158,417,937
178,220,179
198,022,430
Chromosomal Position (bp)
Chromosome 3

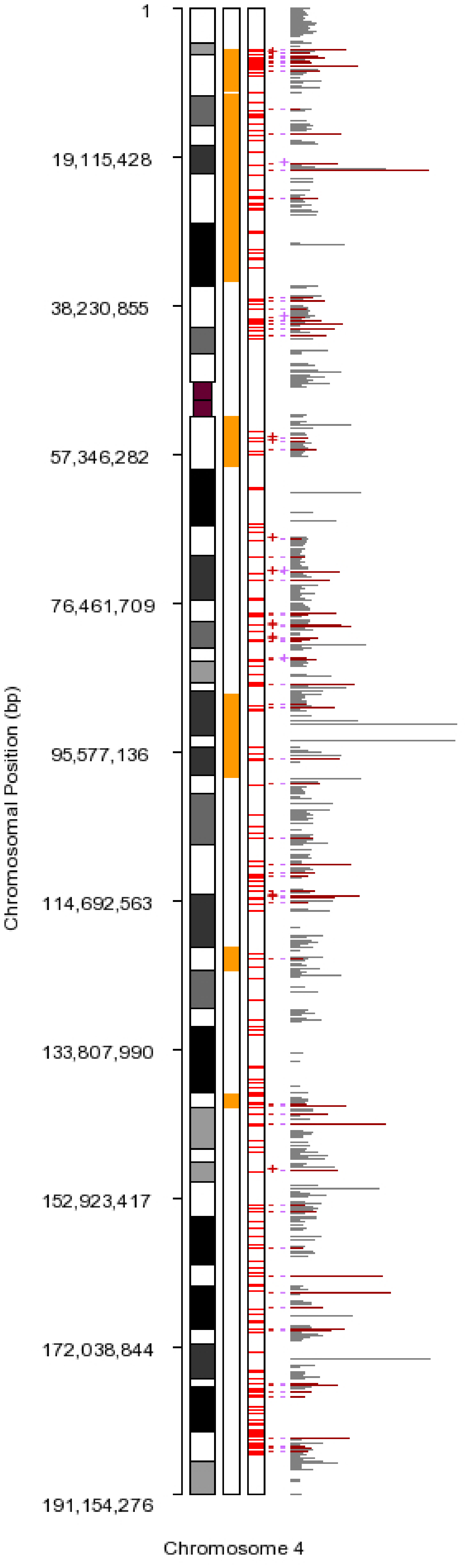

1
19,115,428
38,230,855
57,346,282
76,461,709
95,577,136
114,692,563
133,807,990
152,923,417
172,038,844
191,154,276
Chromosomal Position (bp)
Chromosome 4

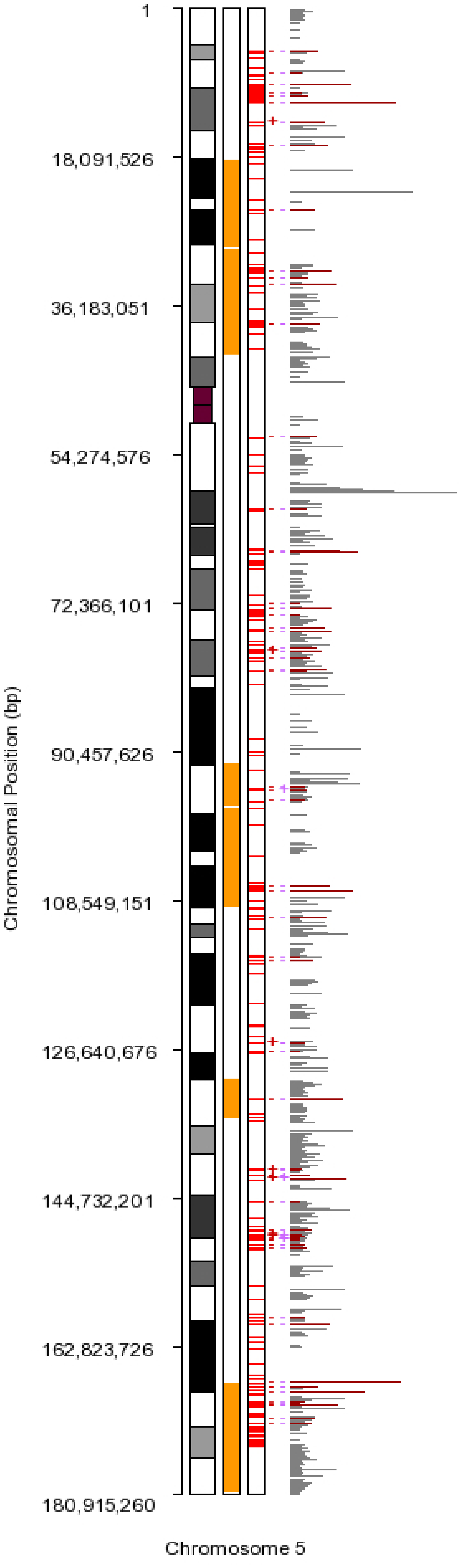
1
18,091,526
36,183,051
54,274,576
72,366,101
90,457,626
108,549,151
126,640,676
144,732,201
162,823,726
180,915,260
Chromosomal Position (bp)
Chromosome 5

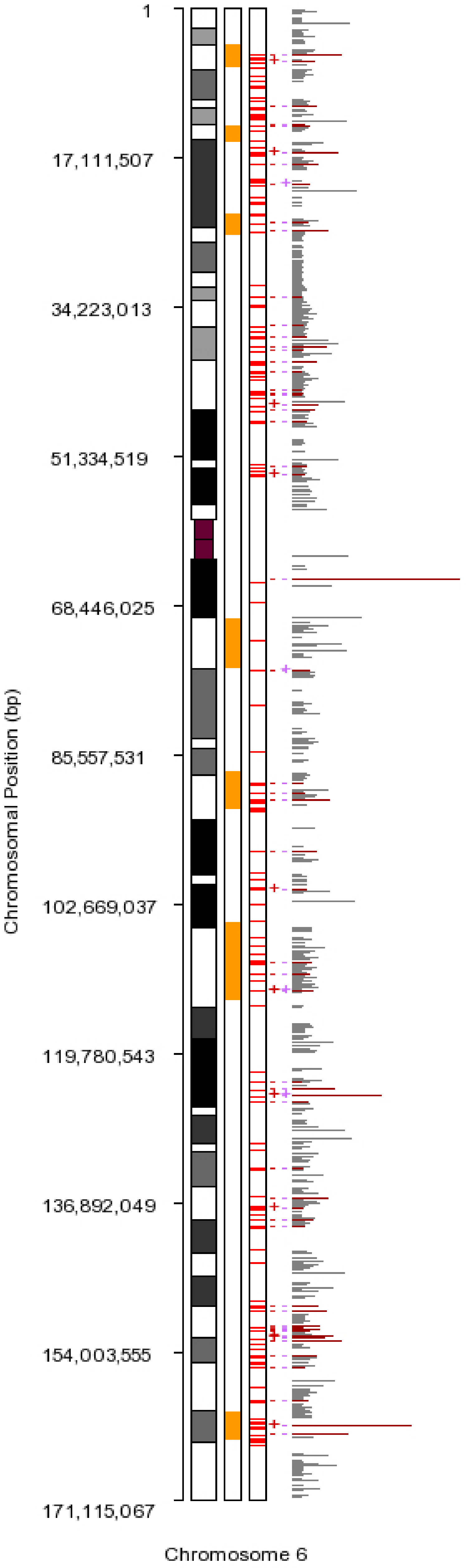
1
17,111,507
34,223,013
51,334,519
68,446,025
85,557,531
102,669,037
119,780,543
136,892,049
154,003,555
171,115,067
Chromosomal Position (bp)
Chromosome 6

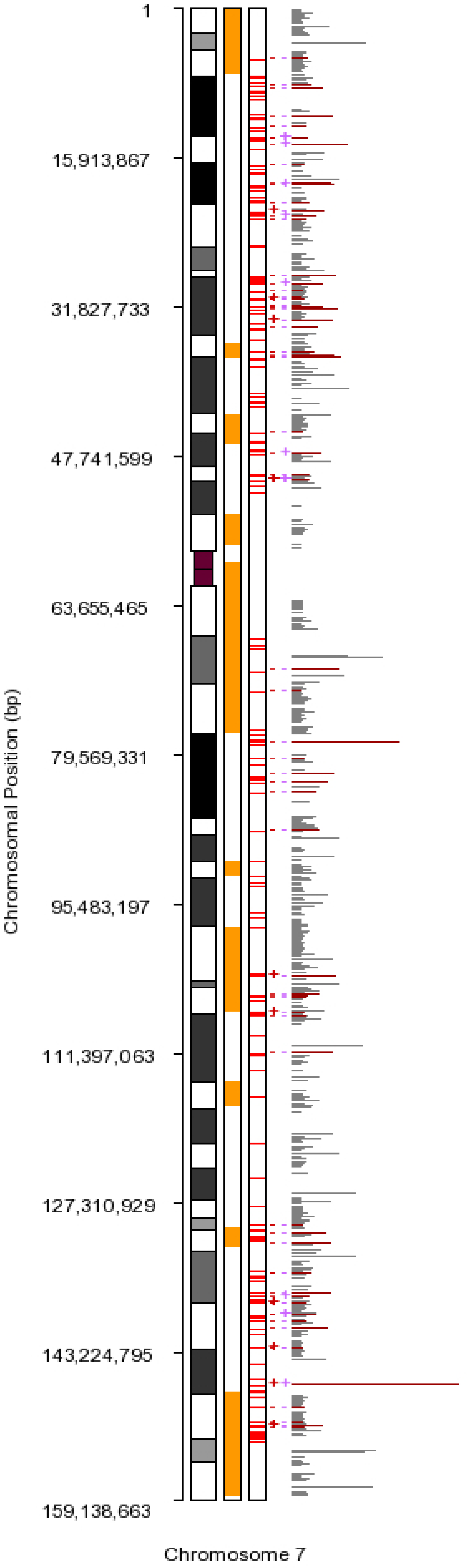

1
15,913,867
31,827,733
47,741,599
63,655,465
79,569,331
95,483,197
111,397,063
127,310,929
143,224,795
159,138,663
Chromosomal Position (bp)
Chromosome 7

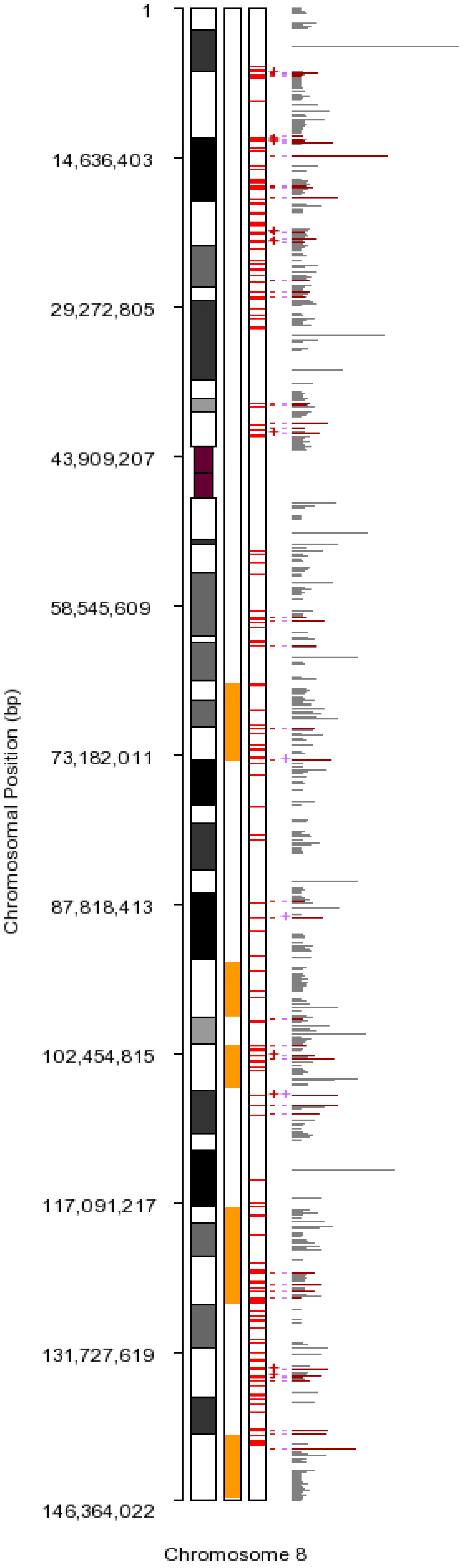

Chromosomal Position (bp)
1
14,636,403
29,272,805
43,909,207
58,545,609
73,182,011
87,818,413
102,454,815
117,091,217
131,727,619
146,364,022
Chromosome 8

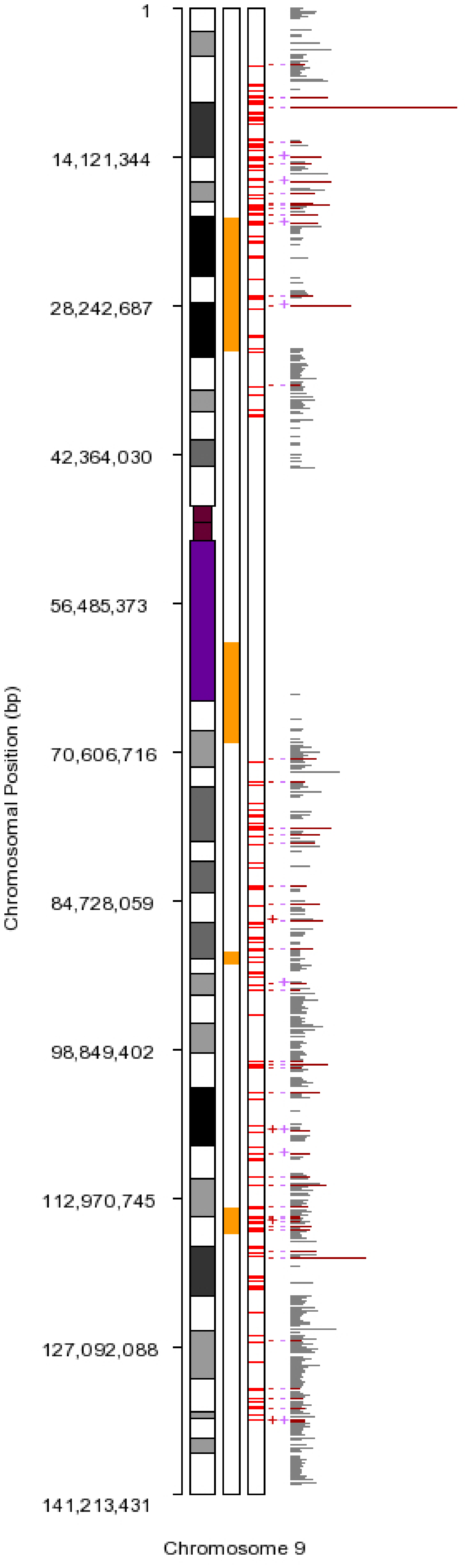

1
14,121,344
28,242,687
42,364,030
56,485,373
70,606,716
84,728,059
98,849,402
112,970,745
127,092,088
141,213,431
Chromosomal Position (bp)
Chromosome 9

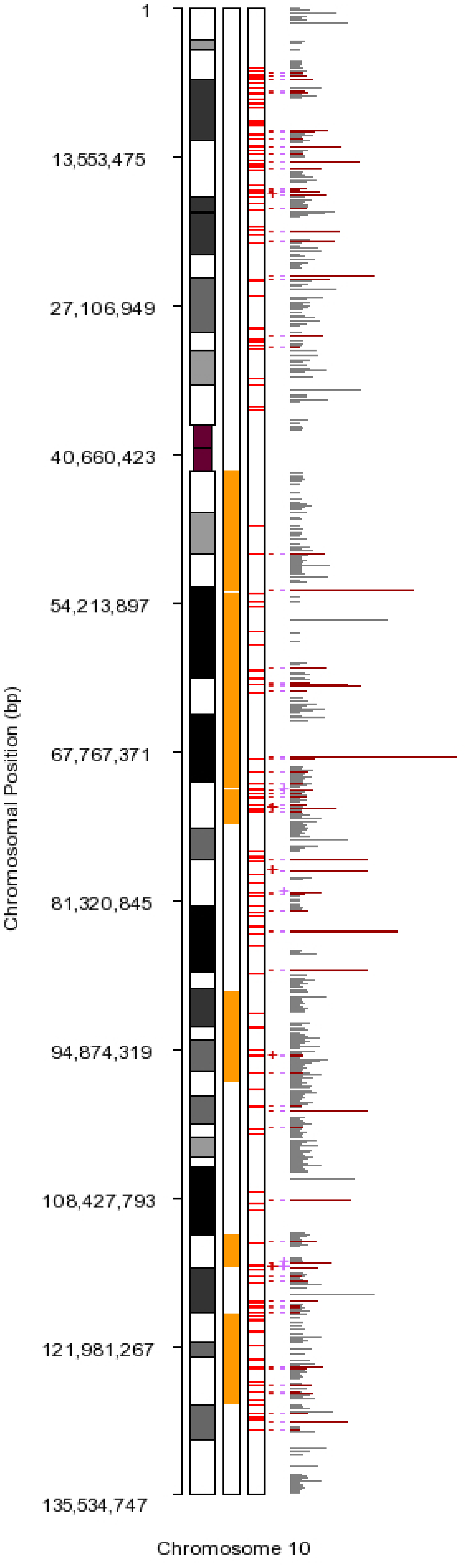
1
13,553,475
27,106,949
40,660,423
54,213,897
67,767,371
81,320,845
94,874,319
108,427,793
121,981,267
135,534,747
Chromosomal Position (bp)
Chromosome 10

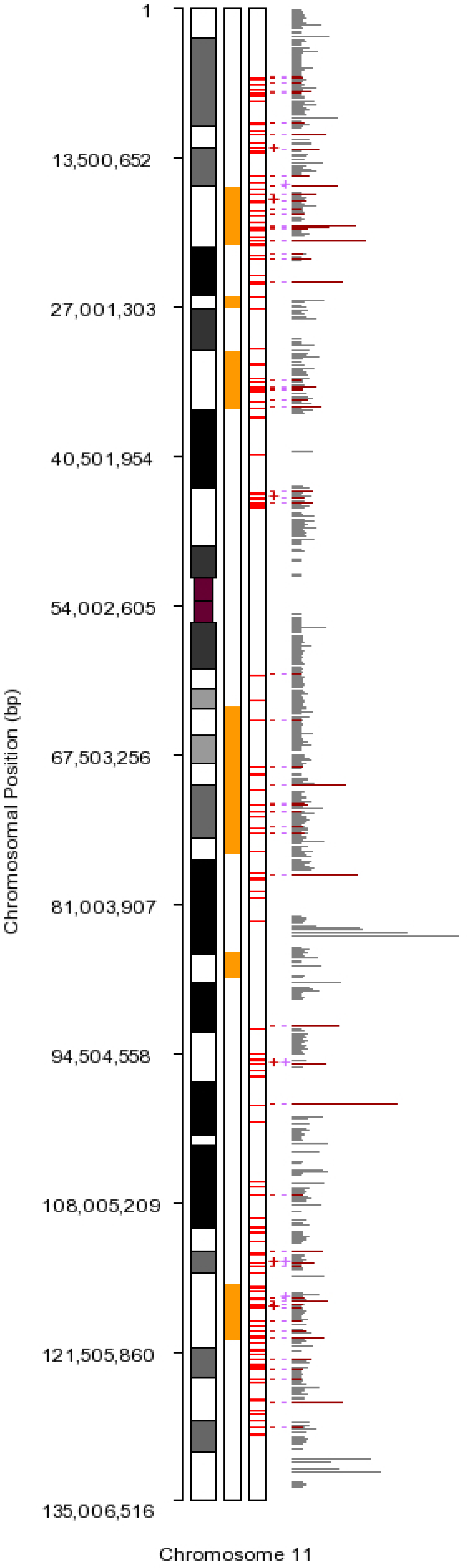

1
13,500,652
27,001,303
40,501,954
54,002,605
67,503,256
81,003,907
94,504,558
108,005,209
121,505,860
135,006,516
Chromosomal Position (bp)
Chromosome 11

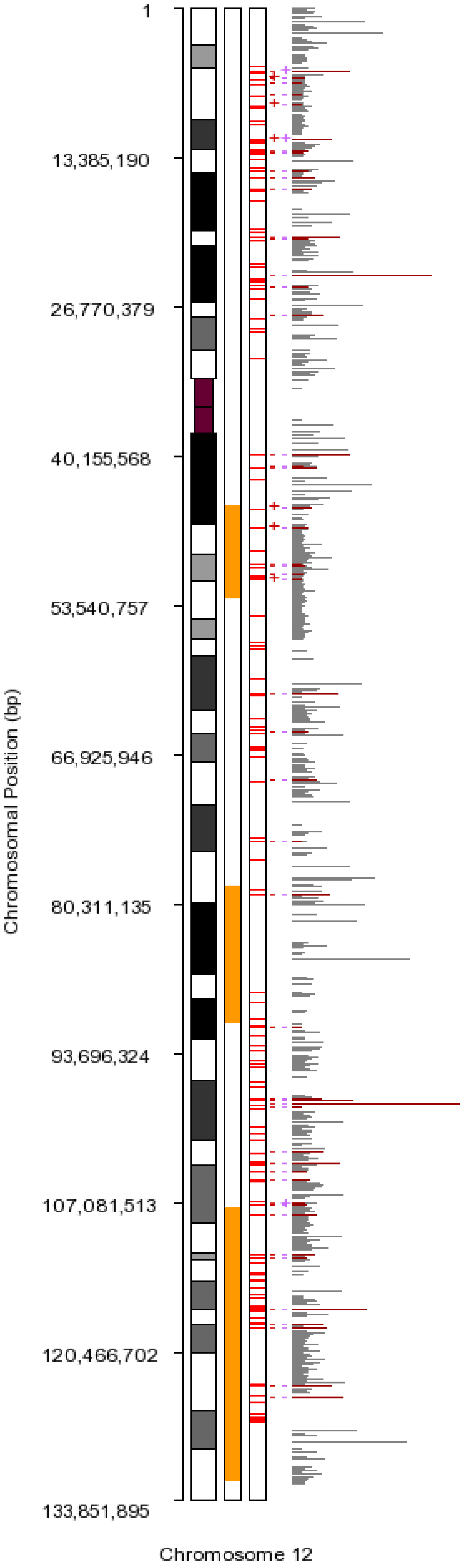
Chromosomal Position (bp)
1
13,385,190
26,770,379
40,155,568
53,540,757
66,925,946
80,311,135
93,696,324
107,081,513
120,466,702
133,851,895
Chromosome 12

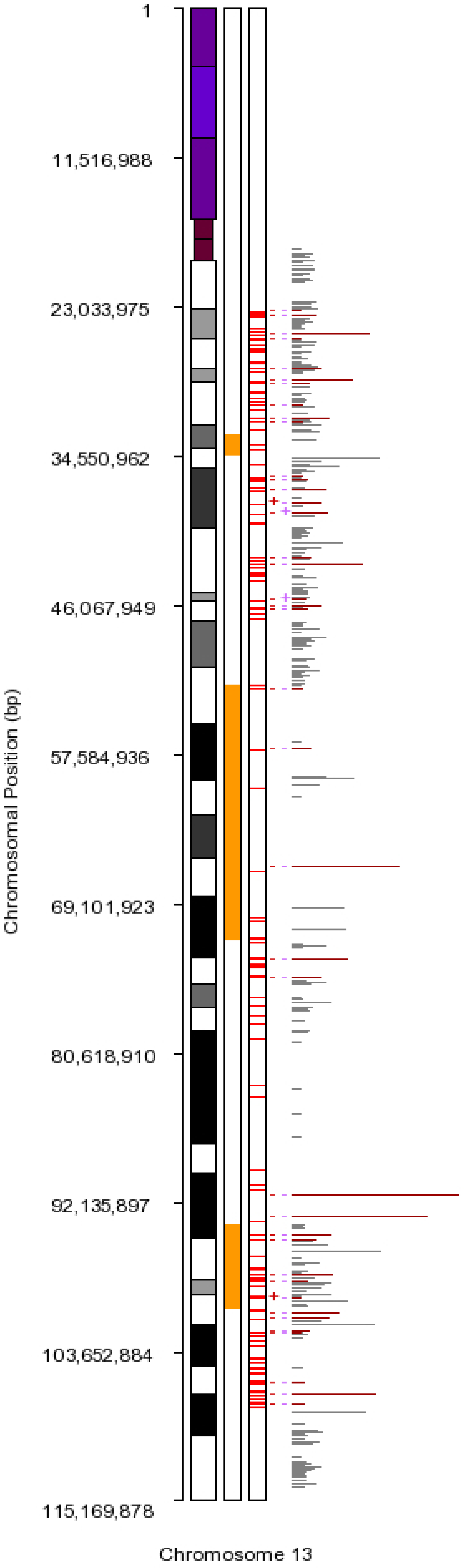
1
11,516,988
23,033,975
34,550,962
46,067,949
57,584,936
69,101,923
80,618,910
92,135,897
103,652,884
115,169,878
Chromosomal Position (bp)
Chromosome 13

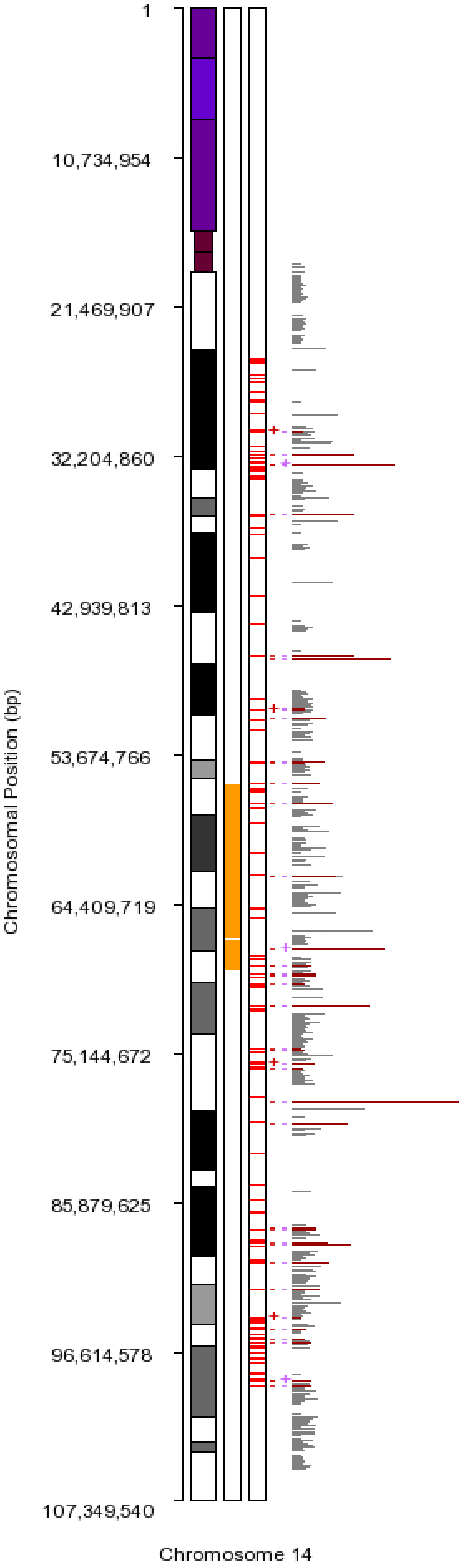
1
10,734,954
21,469,907
32,204,860
42,939,813
53,674,766
64,409,719
75,144,672
85,879,625
96,614,578
107,349,540
Chromosomal Position (bp)
Chromosome 14

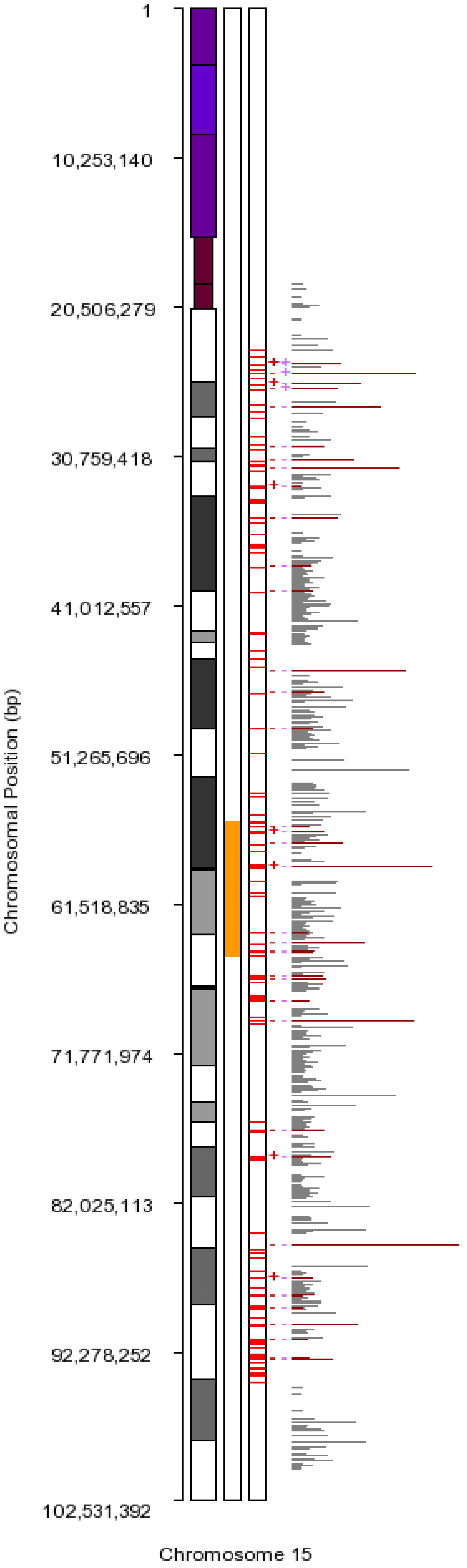

1
10,253,140
20,506,279
30,759,418
41,012,557
51,265,696
61,518,835
71,771,974
82,025,113
92,278,252
102,531,392
Chromosomal Position (bp)
Chromosome 15

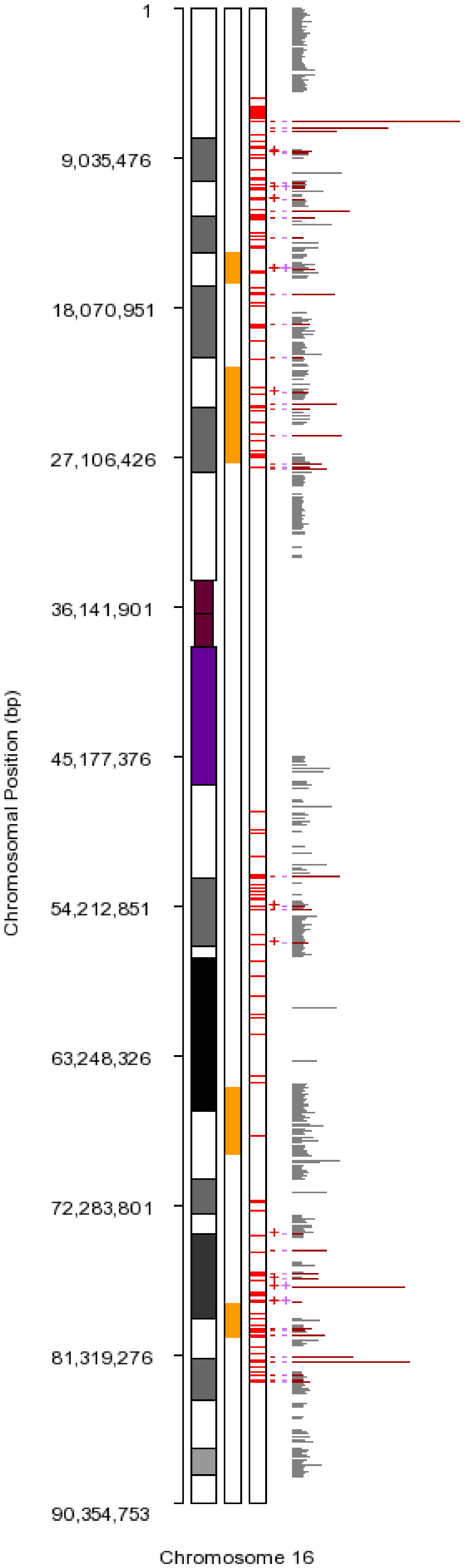
1
9,035,476
18,070,951
27,106,426
36,141,901
45,177,376
54,212,851
63,248,326
72,283,801
81,319,276
90,354,753
Chromosomal Position (bp)
Chromosome 16

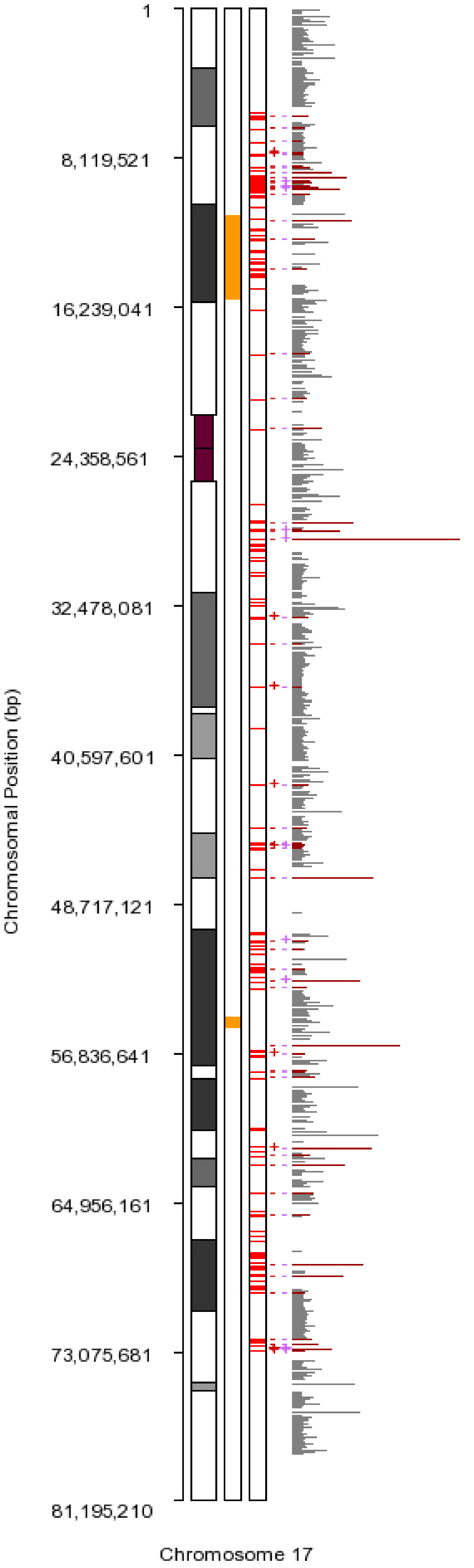
1
8,119,521
16,239,041
24,358,561
32,478,081
40,597,601
48,717,121
56,836,641
64,956,161
73,075,681
81,195,210
Chromosomal Position (bp)
Chromosome 17

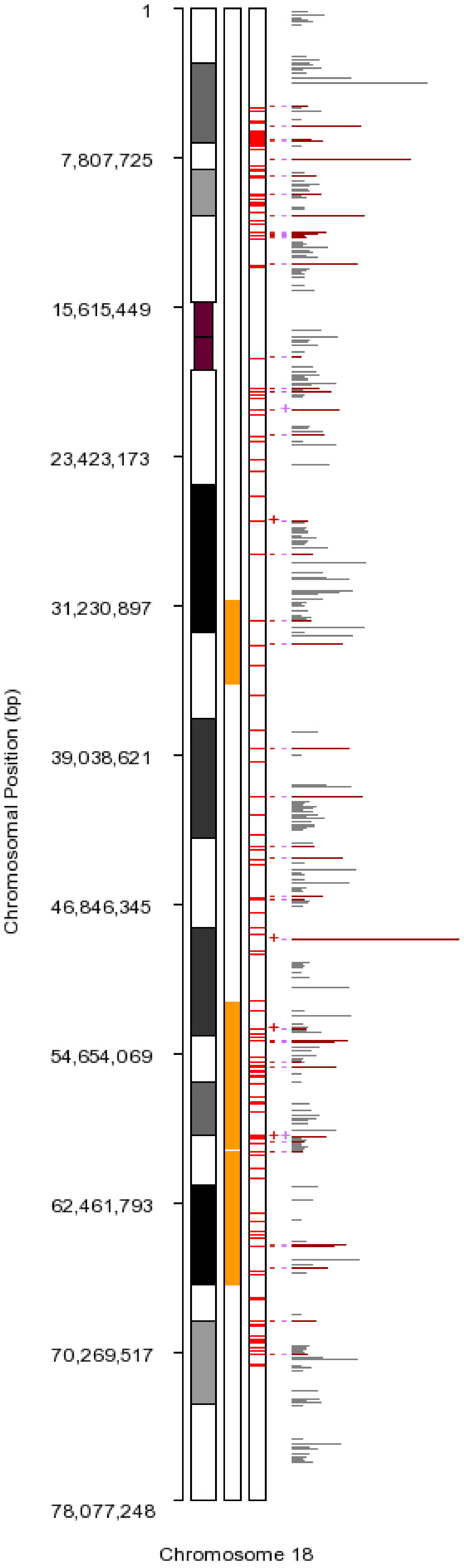

1
7,807,725
15,615,449
23,423,173
31,230,897
39,038,621
46,846,345
54,654,069
62,461,793
70,269,517
78,077,248
Chromosomal Position (bp)
Chromosome 18

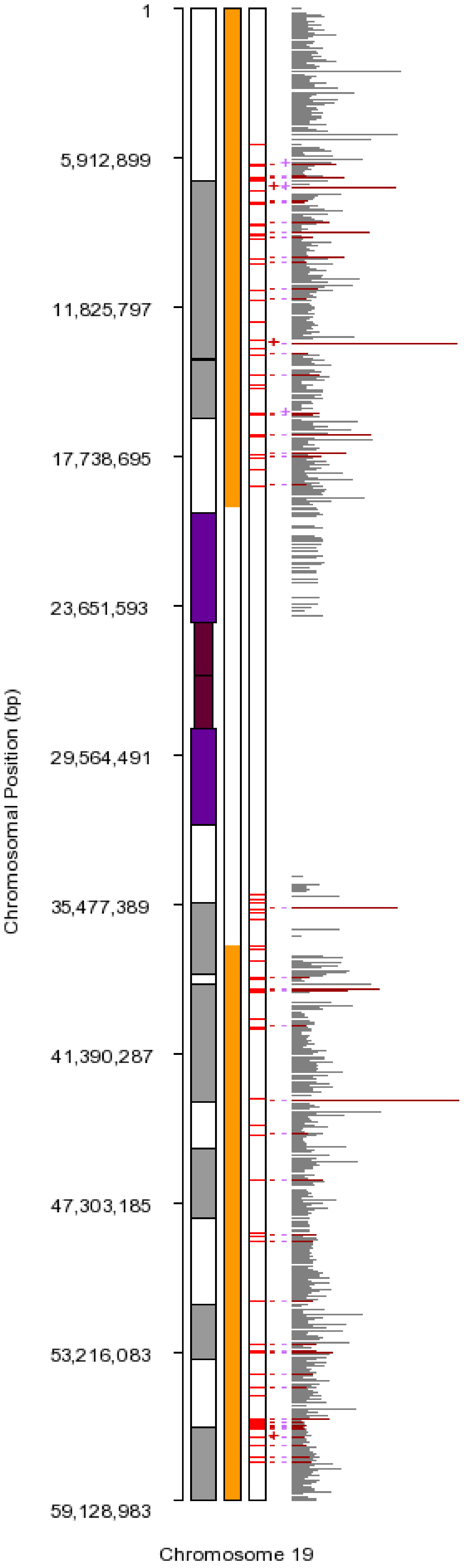

1
5,912,899
11,825,797
17,738,695
23,651,593
29,564,491
35,477,389
41,390,287
47,303,185
53,216,083
59,128,983
Chromosomal Position (bp)
Chromosome 19

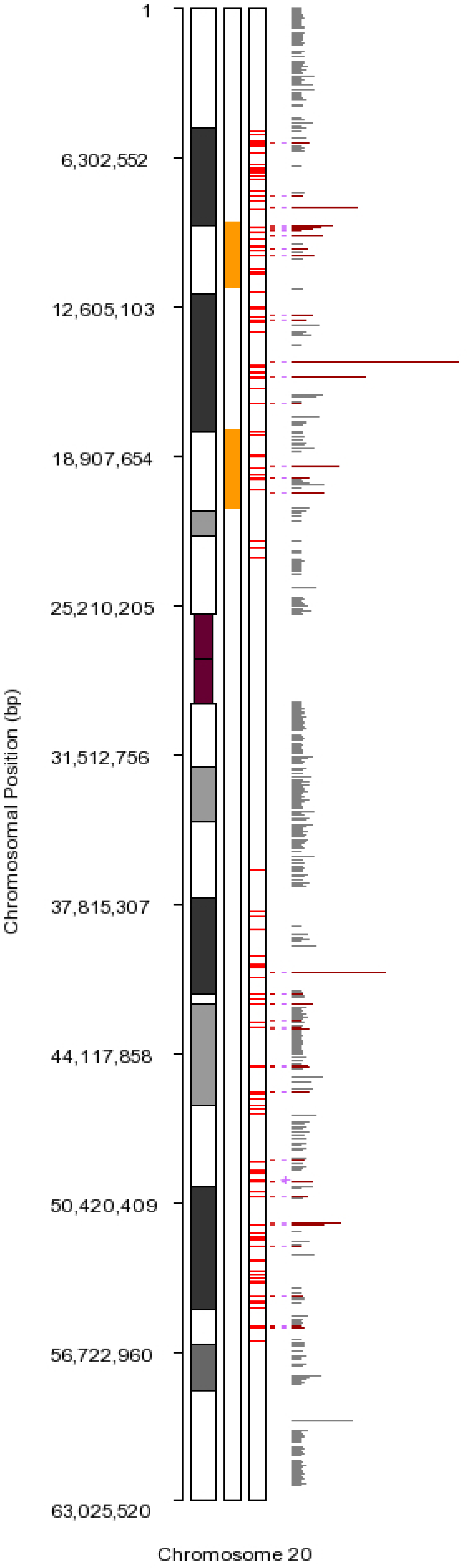
1
6,302,552
12,605,103
18,907,654
25,210,205
31,512,756
37,815,307
44,117,858
50,420,409
56,722,960
63,025,520
Chromosomal Position (bp)
Chromosome 20

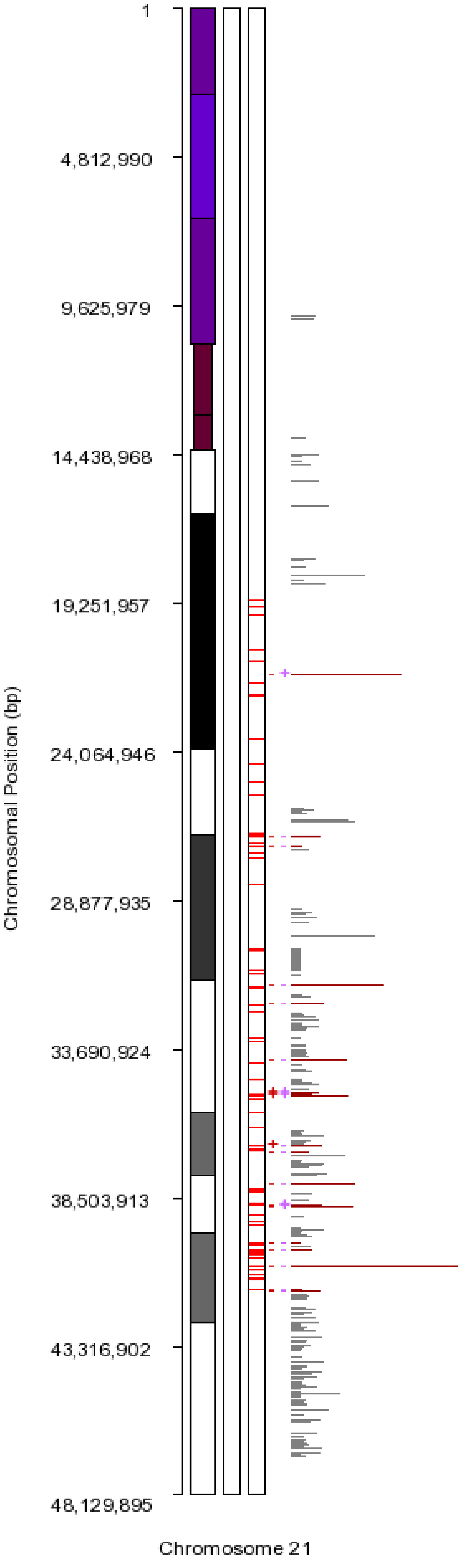
1
4,812,990
9,625,979
14,438,968
19,251,957
24,064,946
28,877,935
33,690,924
38,503,913
43,316,902
48,129,895
Chromosomal Position (bp)
Chromosome 21

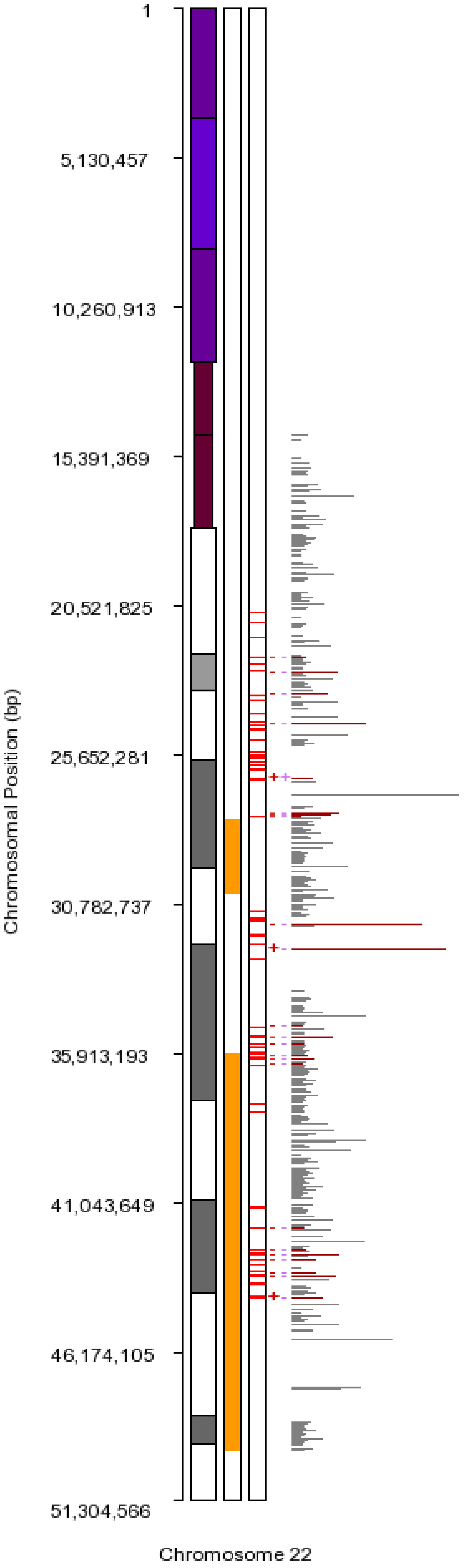
1
5,130,457
10,260,913
15,391,369
20,521,825
25,652,281
30,782,737
35,913,193
41,043,649
46,174,105
51,304,566
Chromosomal Position (bp)
Chromosome 22
